# Experimental Evolution of Metabolic Dependency in Bacteria

**DOI:** 10.1371/journal.pgen.1006364

**Published:** 2016-11-04

**Authors:** Glen D’Souza, Christian Kost

**Affiliations:** 1 Experimental Ecology and Evolution Research Group, Department of Bioorganic Chemistry, Max Planck Institute for Chemical Ecology, Jena, Germany; 2 Department of Ecology, School of Biology/Chemistry, Osnabrück University, Osnabrück, Germany; Uppsala University, SWEDEN

## Abstract

Bacteria frequently lose biosynthetic genes, thus making them dependent on an environmental uptake of the corresponding metabolite. Despite the ubiquity of this ‘*genome streamlining*’, it is generally unclear whether the concomitant loss of biosynthetic functions is favored by natural selection or rather caused by random genetic drift. Here we demonstrate experimentally that a loss of metabolic functions is strongly selected for when the corresponding metabolites can be derived from the environment. Serially propagating replicate populations of the bacterium *Escherichia coli* in amino acid-containing environments revealed that auxotrophic genotypes rapidly evolved in less than 2,000 generations in almost all replicate populations. Moreover, auxotrophs also evolved in environments lacking amino acids–yet to a much lesser extent. Loss of these biosynthetic functions was due to mutations in both structural and regulatory genes. In competition experiments performed in the presence of amino acids, auxotrophic mutants gained a significant fitness advantage over the evolutionary ancestor, suggesting their emergence was selectively favored. Interestingly, auxotrophic mutants derived amino acids not only via an environmental uptake, but also by cross-feeding from coexisting strains. Our results show that adaptive fitness benefits can favor biosynthetic loss-of-function mutants and drive the establishment of intricate metabolic interactions within microbial communities.

## Author Summary

Bacteria frequently lose seemingly essential genes from their genomes that are required to autonomously biosynthesize building block metabolites such as amino acids. It is generally unclear whether these losses are due to chance events in small populations or favored by selection, because loss-of-function mutants may save production cost when utilizing metabolites from the environment. We discovered that populations of *Escherichia coli* that evolved in amino acid-replete environments rapidly lost the ability to autonomously produce several amino acids, which was beneficial when amino acids were present in the environment. Interestingly, these mutants derived amino acids not just from the growth medium, but also from other, co-occurring strains. Our findings show that nutrient-containing environments drive the loss of biosynthetic genes from bacterial genomes and facilitate the establishment of metabolic cross-feeding interactions among bacteria.

## Introduction

Bacterial genomes are highly dynamic in terms of both size and composition [1]. The extensive variation in gene repertoires that characterizes prokaryotic genomes can be caused by genome expansion via horizontal gene transfer and gene duplication or, alternatively, contraction due to gene loss. Interestingly, comparative analyses have provided evidence that gene loss may in fact be quantitatively more important for determining the size of prokaryotic genomes than the gain of new genetic information [1–3]. Indeed, as sequencing technologies improve, more and more microorganisms are being discovered that feature tremendously small genomes [4]; some of which are even smaller than the suggested minimal genome size for cellular life of ~300 kb [5]. Analyzing the genetic content of these reduced genomes revealed—besides a lack of dispensable elements [6]—also the elimination of seemingly essential biosynthetic functions. For example, reconstructing metabolic networks from sequence data to predict the phenotype of the focal organism unraveled that the majority of bacterial genomes analyzed lacked the biosynthetic capability to produce several essential building block metabolites such as amino acids, vitamins, or even nucleobases [7–10]. Surprisingly, the list of genotypes that cannot produce certain metabolites autonomously (hereafter: *auxotrophic genotypes*) does not only include host-associated bacteria such as pathogens [11] or endosymbionts [12–14], which potentially obtain the required metabolites from their host’s cytoplasm, but also free-living bacteria such as *Prochlorococcus* and *Pelagibacter* [15, 16] that are known to mainly inhabit nutrient-poor environments. The ubiquity of biosynthetic loss-of-function mutations in bacteria that inhabit ecologically disparate environments begs an explanation: Which evolutionary mechanisms have favored a loss of biosynthetic genes over metabolic autonomy in these bacteria?

Two main hypotheses have been put forward to explain these striking observations. First, genetic drift may drive gene loss in bacteria that are obligately associated with eukaryotic hosts. These bacteria experience nutrient-rich and rather constant environmental conditions [17] and frequently undergo reductions in population sizes during host-to-host transmission. As a consequence of these periodic population bottlenecks, the effects of drift may override those of selection [2]. A lack or a drastically reduced frequency of recombination may further accelerate the fixation of non-beneficial or deleterious mutations [12]. This hypothesis is mainly supported by evidence stemming from comparative genomic analyses [18–20]. In addition, selection experiments, in which bacterial populations were repeatedly subjected to single-cell bottlenecks, resulted in bacteria with strikingly reduced genomes [21].

The second main hypothesis that has been proposed to account for the ‘*streamlining*’ of bacterial genomes is that natural selection favors loss-of-function mutants in environments, in which the gene is no longer required [2, 22–24]. This line of reasoning has been mainly applied to bacteria with free-living lifestyles or those that face nutrient-poor conditions. The large effective population size bacteria experience in these environments likely increases the efficacy of selection. For instance, it has been previously shown that advantageous mutations occur frequently in experimentally evolved bacterial populations of large effective population sizes [25]. As a consequence, loss-of-function mutations or gene deletions that increase a cell’s fitness are more likely to fix in the population [2]. Adaptive benefits of losing genes may stem, for example, from an increased cellular economization [26] or a saving of production costs, when certain metabolites can be derived from the environment [8, 23]. Indeed, previous studies that compared the Darwinian fitness of engineered mutants lacking the ability to biosynthesize certain metabolites to non-mutated wild type cells revealed that metabolic auxotrophies can be beneficial, when the required biosynthetic product is sufficiently available in the environment [7, 8, 22, 23]. Furthermore, fitness-increasing deletion mutations have also been observed in bacterial populations adapting to environments, in which the lost functions were not required for survival [27, 28].

Even though these studies suggest that selection can possibly account for the commonly observed loss of biosynthetic genes from bacterial genomes, the frequencies with which these mutations arise in nutrient-containing environments as well as the fitness effects they exert on the corresponding mutants remain generally unclear. Therefore, direct experimental evidence for natural selection driving the loss of biosynthetic functions, and thus facilitating a metabolic adaptation to the current nutrient environment, is lacking.

To unravel whether fitness advantages can indeed drive the loss of biosynthetic functions from bacterial genomes in nutrient-containing environments, we serially propagated eight replicate populations of the initially metabolically autonomous (hereafter: *prototrophic*) bacterium *Escherichia coli* in amino acid-replete (hereafter: *AA regime*) or -deficient environments (hereafter: *non-AA regime*). After 2,000 generations of evolution in the presence of 20 amino acids, 75% of the experimental populations evolved amino acid auxotrophies–on average for 10 amino acids per strain. Surprisingly, auxotrophic mutants also evolved in the non-amino acid environment, albeit at lower frequencies than in the amino acid-containing environment. The evolution of metabolic auxotrophies was adaptive when amino acids were present in the environment and–surprisingly–also when other co-occurring genotypes could provide the required amino acids. A genomic analysis of derived auxotrophic genotypes revealed that distinct genetic changes in both *structural* and *regulatory* genes caused the adaptive loss or deactivation of biosynthetic functions. Our analysis indicates that adaptive advantages can drive the evolution of metabolic auxotrophies in bacteria and thus foster their obligate dependency on the biotic and abiotic environment.

## Results

### The nutritional environment affects the evolutionary trajectories of evolving populations

**Fig 1:**
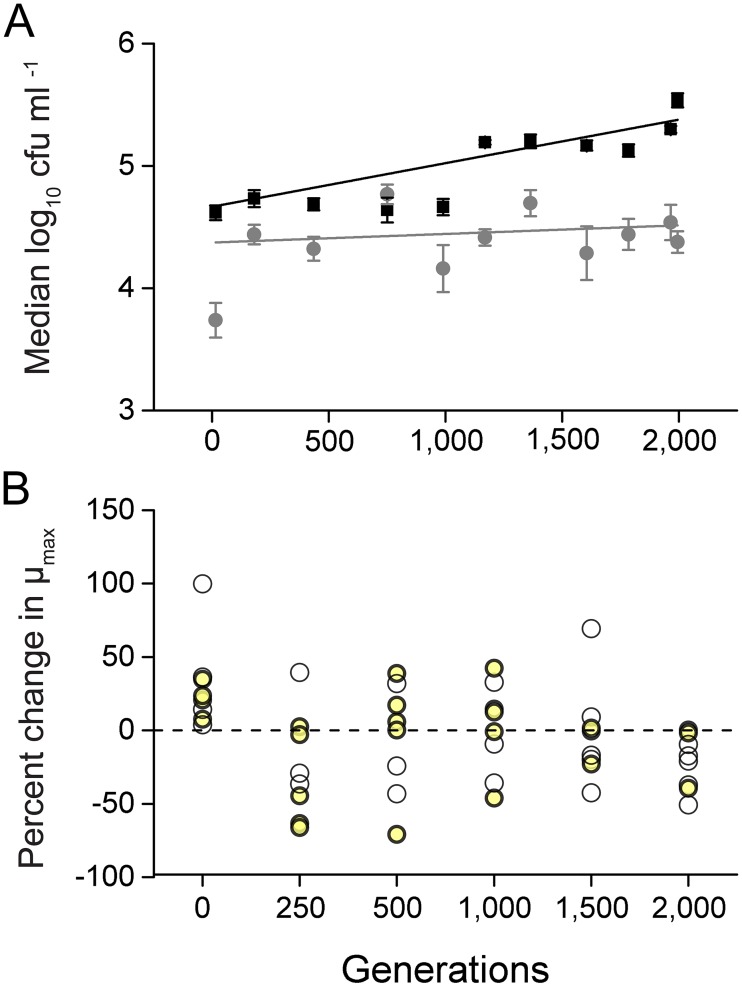
In the presence of amino acids, populations evolved an increased cell density and amino acid-dependency of their growth. (A) Median cell density of the populations that evolved in the presence (black boxes) or absence of AAs (grey circles) over the course of the evolution experiment. Each data point represents the median cell density (± 95% confidence interval) of eight replicate populations that are displayed as the log_10_ of the number of colony forming units per ml (CFU ml^-1^) at different time points of the experiment. The black and grey lines represent the exponential fit of the individual points (linear regression of fitted lines: P<0.05, n = 8). (B) Amino acid dependency of growth expressed as the mean difference in percentage between the maximal growth rates the AA-evolved populations achieved under AA-replete conditions and the growth rates, they achieved under unsupplemented conditions. Positive values indicate populations grow better in the absence of AAs, while negative values point to a growth advantage when AAs are present. Each circle represents the mean value of the corresponding replicate population and yellow circles highlight those populations, whose growth differed significantly between the two environments (FDR corrected paired samples t-test: P<0.05, n = 4).

To determine how the growth environment can affect the evolution of adapting bacterial populations, eight replicate populations of *Escherichia coli* were serially propagated by daily transfer of 1,000 cells in minimal medium that did or did not contain all of 20 different amino acids (AAs). Quantifying the cell density that each population achieved at different time points of the evolution experiment as the number of colony forming units (CFUs) ml^-1^ indicated that the mere presence of AAs already benefitted the ancestral genotype as indicated by a significantly increased productivity relative to AA-deficient conditions (independent samples t-test: P<0.05, n = 8, Fig 1A). This initial pattern remained consistent as these populations were further propagated, resulting in a significantly increased slope of the populations that evolved in the presence of AAs as compared to populations that were selected in unsupplemented minimal medium (independent sample t-test: P<0.05, n = 8, Fig 1A). In other words, the presence of nutrients significantly increased the rate of increase in cell density. Given that the size of a bacterial population affects its rate of adaptation via influencing the size of fitness advantage of fixing beneficial mutations [29], reaching an increased cell density in the presence of AAs likely sped up adaptation in these evolving populations.

To determine whether the increased cell density of populations that evolved in the presence of AAs was indeed due to the increased availability of nutrients, the growth rates (μ_max_ h^-1^) the focal populations achieved in the presence (i.e. the selection regime) of AAs was subtracted from the growth rates the same populations achieved under AA-deficient conditions and the percent change in growth rates was calculated. The resulting value (percent change) was significantly greater than zero for 4 of 8 of the ancestral populations (false discovery rate (FDR)-corrected paired samples t-test: P<0.05, n = 4, Fig 1B), indicating that the exponential growth of the evolutionary ancestor was inhibited in the presence of AAs. However, over the course of evolution, an omission of amino acids from the test medium resulted in a decline of growth rates for most of the replicate populations to the point that at 2,000 generations, seven out of eight populations showed significantly reduced growth rates in the absence of AAs (FDR-corrected paired samples t-test: P<0.05, n = 4, Fig 1B).

In summary, analyzing the evolution experiment on a population level revealed that the rate, with which populations of *E*. *coli* increased in cell density, was increased in the presence of AAs. Moreover, the growth of AA-evolved populations became increasingly dependent on the presence of AAs over the course of the evolution experiment.

### Amino acid auxotrophies rapidly evolved in amino acid-containing environments

**Fig 2:**
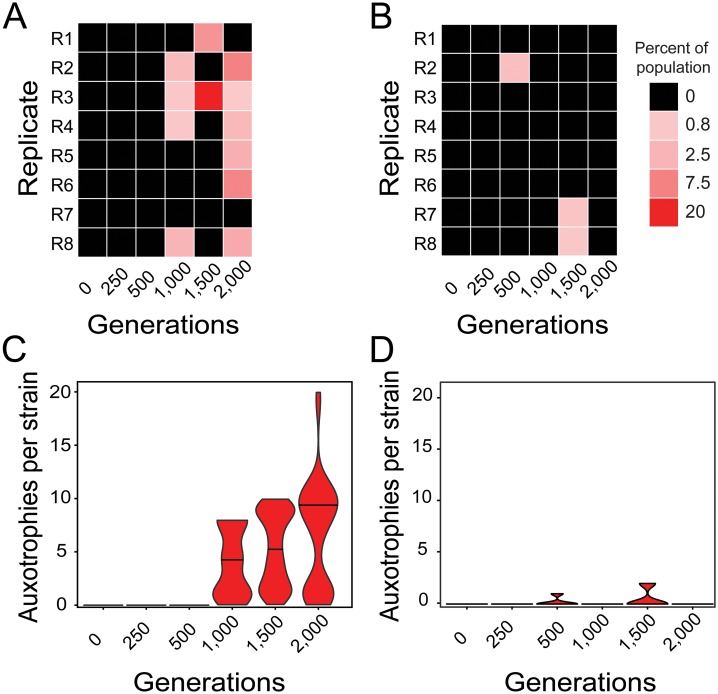
Rapid evolution of amino acid auxotrophies. (A,B) Heatmaps depict the population-level frequency AA-auxotrophic genotypes reached per 1,000 CFUs analyzed when evolving in (A) the presence or (B) the absence of AAs. Rows represent replicate populations (R1-R8) and columns are different evolutionary time points. Different colors indicate the frequencies of auxotrophic strains detected per population (see legend). (C,D) Distribution of the number of AA auxotrophies identified per auxotrophic strain isolated from (C) the AA regime and (D) the non-AA regime at different evolutionary time points. Violin plots are scaled to the same maximum width and lines within plots indicate the medians of the distributions.

One possibility to explain the AA-dependent increase in the productivity of populations that evolved in AA-containing environments could be the emergence of AA auxotrophic genotypes that benefitted from utilizing environmentally available AAs. These strains would be unable to grow in the absence of AAs, yet can grow when AAs are present [8]. To determine whether and to which extent auxotrophic genotypes evolved in both selection regimes, 1,000 colonies of each replicate population from different evolutionary time points were screened for the presence of auxotrophic genotypes. After 0, 250, and 500 generations, no auxotrophic CFU was detected in any of the populations that evolved in the presence of AAs (Fig 2A). However, when populations from later time points were considered, 50% (1,000 generations), 25% (1,500 gens.), and 75% (2,000 gens.) of the AA-evolved populations contained auxotrophic genotypes (Fig 2A). The proportion of auxotrophic genotypes detected in these populations ranged between 0.8% and 2.5% after 1,000 generations, between 5.7% and 20% after 1,500 generations, and between 0.6% to 7.5% after 2,000 generations of evolution in the AA-containing environment (Fig 2A).

Intriguingly, three of the eight populations that evolved without an external supply of AAs also featured AA auxotrophic strains (Fig 2B): Replicate population 3, which had evolved for 500 generations contained nearly 2% of auxotrophic genotypes, while replicate populations 7 and 8 comprised approximately 1% of auxotrophic strains after 1,500 generations of evolution (Fig 2B). Detecting auxotrophic genotypes in populations that evolved in the absence of AAs is surprising and suggests that these loss-of-function mutants likely obtained the AAs they required for growth from the coexisting prototrophic genotypes, which were present in high frequencies.

A striking pattern that arose in both selection regimes was the dynamics that characterized the emergence of auxotrophic genotypes. Even though the number of auxotrophic genotypes generally increased over evolutionary time, their distribution and abundance within replicate populations showed a high degree of fluctuation around the detection limit of 1,000 cells (Fig 2). For instance, replicate population 1, which has evolved in the presence of AAs, contained 5.7% auxotrophic strains after 1,500 generations, but no auxotrophic strain was detectable after 2,000 generations. However, given that a frequency change of 1% corresponds to at least 10^4^ auxotrophic cells, the observed fluctuations are significant on a population-level. Moreover, the fact that their frequency rose to detectable levels (≥10^4^ cells) implies these mutants were likely selectively favored.

Analyzing the number of different metabolic auxotrophies that were found on a genotype-level revealed that strains isolated from the AA-containing environment were generally impaired in the biosynthesis of more than four different AAs simultaneously, while auxotrophs that evolved in the AA-deficient environment depended on average on only one or two different AAs (Pearson’s chi square test: P<0.05, n = 483 (AA regime) and 37 (non-AA regime), Fig 2C and 2D; S1 Table). Some of the strains, which have been isolated after 2,000 generations of evolution in the AA-containing environment, even required all 20 AAs for growth (Fig 2C). Auxotrophic types that emerged in individual populations after 2,000 generations of adapting to an AA-containing environment showed a striking congruence in the identity of amino acid auxotrophies that evolved in these populations (S1 Fig), suggesting adaptive advantages likely drove this pattern.

Taken together, these results demonstrate that AA auxotrophies evolved in both selection regimes, yet to a significantly larger extent when AAs were present in the environment. The finding that the frequency of auxotrophic strains increased under both conditions to detectable levels implies that these loss-of-function mutants were likely selectively favored.

### Evolved auxotrophies are adaptive when amino acids are environmentally available

**Fig 3:**
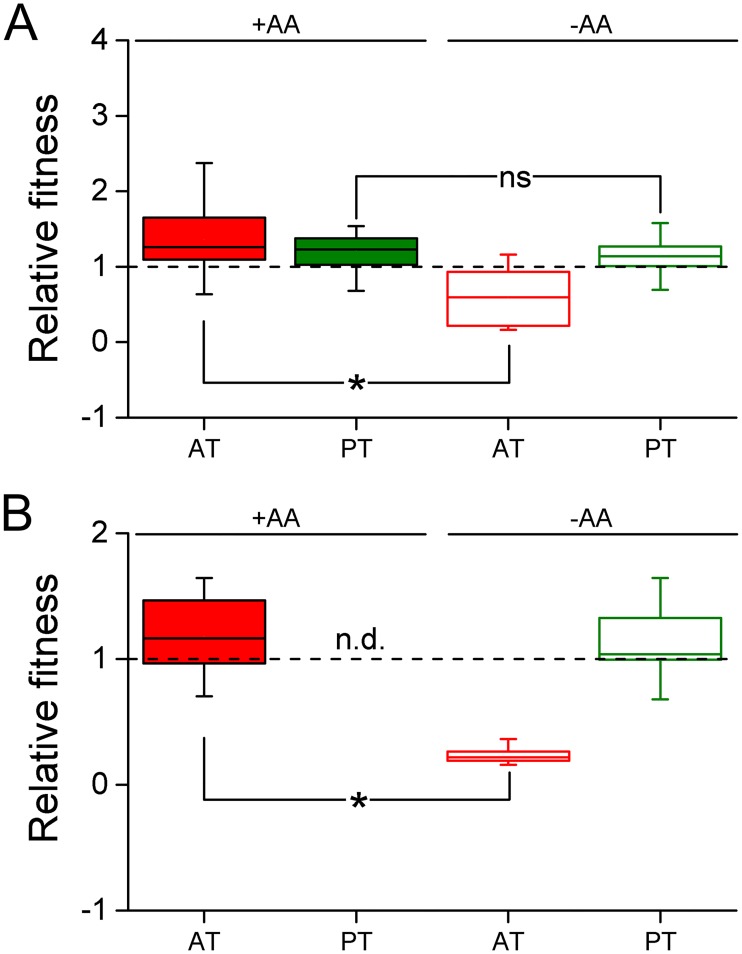
Fitness advantage of auxotrophs, but not prototrophs is AA-dependent. Competitive fitness of derived auxotrophs (AT, red boxes) or prototrophs (PT, green boxes) that evolved in (A) the presence or (B) the absence of AAs relative to their evolutionary ancestor was determined in the presence (filled boxes) or absence (empty boxes) of AAs. Relative fitness is the ratio of Malthusian parameters of each group of genotypes and the dashed line represents the fitness of the evolutionary ancestor. Asterisks indicate significant fitness differences between experimental conditions (independent samples t-test: P<0.05, n = 4 for 28 derived auxotrophic or prototrophic strains from the AA regime and n = 4 for 3 derived auxotrophic or prototrophic strains of each strain from the non-AA regime), while ‘ns’ denotes non-significant differences. The relative fitness of both derived types (AT and PT) differs significantly from that of the ancestor under both conditions considered (independent samples test: P<0.05, n = 4). ‘nd’ indicates the corresponding value was not determined. Box plots: medians (horizontal lines within boxes), interquartile range (boxes), and 1.5x-interquartile range (whiskers).

To identify whether the rapid emergence and spread of auxotrophic genotypes was driven by selective advantages that resulted from the loss of biosynthetic functions, auxotrophic and prototrophic genotypes that evolved in either the absence or presence of all AAs were individually competed against their common evolutionary ancestor. Determining competitive fitness in this way revealed that auxotrophic strains, which evolved in the presence of AAs, were significantly fitter than their evolutionary ancestor when all 20 AAs were present in the environment (i.e. the selection regime) (FDR-corrected independent samples t-test: P<0.05, n = 4 for each of 28 the auxotrophic strains in S1 Table, Fig 3A). However, when AAs were omitted, these auxotrophic genotypes were significantly less fit than the ancestral genotype (FDR-corrected independent samples t-test: P>0.05, n = 4 for each of the 28 auxotrophic strains isolated from the AA regime, S1 Table, Fig 3A). This observation implies that auxotrophic genotypes increased in frequency, because they gained an adaptive advantage when AAs were present in the environment. In contrast, the evolutionary success of derived prototrophs was independent of an environmental availability of AAs, as indicated by the finding that their fitness was significantly increased over ancestral levels independent of whether or not AAs were present in the environment (FDR-corrected paired samples t-test: P>0.05, n = 4 for each of the 28 prototrophic strains in that coexisted with the auxotrophic strains, S1 Table, Fig 3A).

A qualitatively similar picture emerged when the derived strains of the non-AA regime were analyzed: Supplementing AAs to the growth medium resulted in an increased fitness of auxotrophic genotypes relative to their prototrophic ancestor (FDR-corrected independent sample t-test: P<0.05, n = 4 for 3 derived auxotrophic strains, Fig 3B). However, under unsupplemented conditions (i.e. the selection regime), auxotrophs were less fit than the ancestor (FDR-corrected independent samples t-test: P<0.05, n = 4 for 3 derived auxotrophic strains, Fig 3B). In line with the above findings, prototrophic genotypes isolated from the derived non-AA populations gained a significant fitness advantage over their ancestor in the absence of AAs (i.e. the selection regime) (FDR-corrected independent samples t-tests: P<0.05, n = 4 for 3 derived prototrophic strains, Fig 3B) that was quantitatively comparable to the advantage AA-evolved prototrophs gained under the same conditions (Fig 3).

Taken together, these findings imply that an environmental availability of AAs favored mutants that have lost the ability to autonomously biosynthesize certain AAs. In contrast, the fitness advantage gained by prototrophic genotypes was independent of the presence of AAs in the environment.

### Auxotrophs evolved a metabolic dependency on coexisting prototrophs

Two findings of the abovementioned experiments beg an explanation. First, AA auxotrophies evolved even when no AAs were present in the environment (Fig 2B). Second, even though AA-evolved auxotrophs were less fit than the ancestor when no AAs were present in the environment (FDR-corrected independent samples t-test: P<0.05, n = 4 for 28 derived auxotrophic strains, Fig 3A), these genotypes still grew to detectable frequencies in the corresponding fitness assays. From where did these auxotrophic genotypes obtain the AAs they needed to grow? A likely source could be the prototrophs that coexisted with the auxotrophic genotypes in the two abovementioned experiments. To test this possibility, auxotrophic genotypes, which were phenotypically dissimilar based on their metabolic auxotrophies and isolated after 2,000 generations (S1 Table), were grown in monoculture, together with the prototroph they coevolved with, or the evolutionary ancestor (both cocultures: 1:1 ratio). This test was performed in either the absence or presence of AAs and the fitness of auxotrophs (i.e. their Malthusian parameter) was determined.

**Fig 4:**
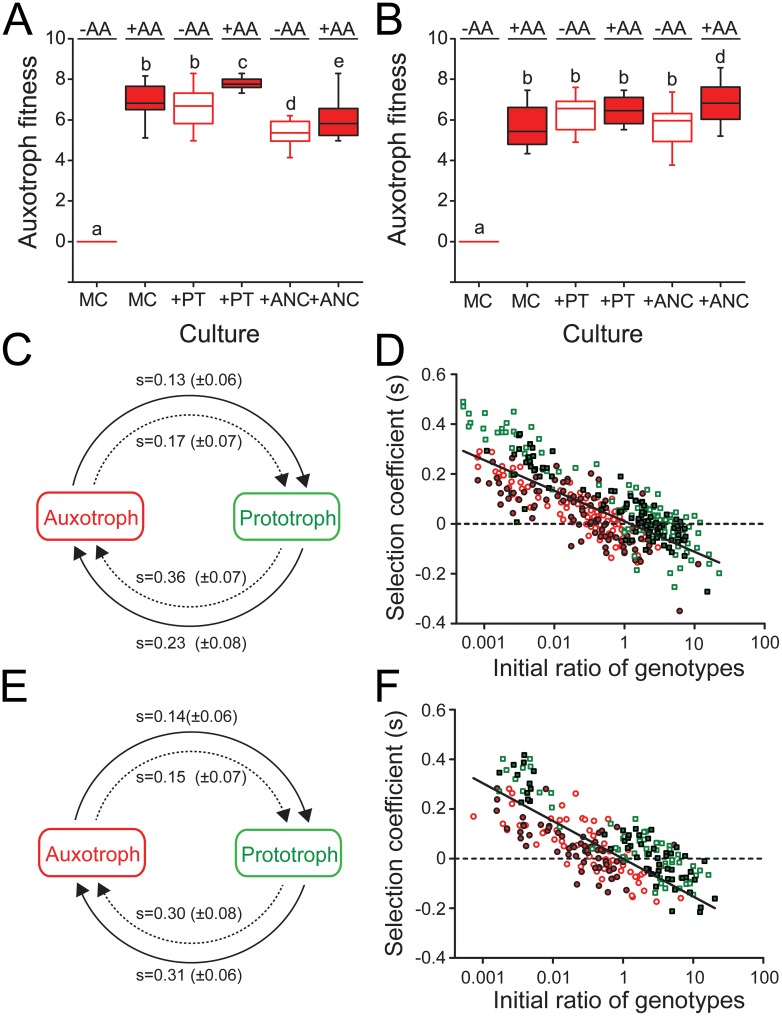
Auxotrophic strains evolved frequency-dependent interactions with coexisting prototrophs. (A,B) Fitness of auxotrophic strains that have been isolated after (A) 2,000 generations of growth under AA-replete or (B) 1,500 generations of growth under AA-deficient conditions expressed as the Malthusian parameter. Growth was determined in monoculture (MC) or in coculture with either a prototrophic strain isolated from the same time-point and population (+PT) or the evolutionary ancestor (+ANC) in the presence (red boxes) or absence of AAs (empty boxes). Different letters indicate significant differences in the auxotrophs’ fitness (one-way ANOVA followed by a LSD post-hoc test: P<0.05, n = 4 for 6 derived auxotrophic strains for the AA regime and n = 4 for 3 derived auxotrophic strains for the non-AA regime). (C-F) Reciprocal invasion-from-rare experiments show the interaction between coevolved auxotrophic (red) and prototrophic (green) genotypes isolated from (C,D) the AA regime or (E,F) the non-AA regimes is stabilized by negative frequency-dependent selection. Competition experiments between auxotrophic and prototrophic strains were initiated at a ratio of 1:100 or 100:1 and subsequently propagated daily for 5 days (i.e. 75 generations). Selection coefficients (s) were determined at the end of every transfer cycle. (C,E) Arrows point from the initially rare invader to the common type (initial ratio: 1:100) that is being invaded in the presence (solid arrows) or absence (dashed arrows) of AAs. Numbers above arrows are mean selection coefficients (± 95% confidence interval) after the first transfer (i.e. 24 h), which in all cases were significantly larger than zero, indicating a successful invasion (one sample t-test: P<0.05, n = 4 for 6 derived auxotrophic or prototrophic strains for the AA regime and n = 3 for 4 derived auxotrophic or prototrophic strains for the non-AA regime). (D,F) Selection coefficients of the auxotrophic (circles) and prototrophic (squares) genotypes after a transfer as a function of its initial ratio at the onset of every growth cycle. The experiment was performed in both the presence (filled symbols) of absence (open symbols) of AAs and the data of the entire experiment (i.e. 5 d) is plotted cumulatively. The solid line represents the linear regression of s on the ratio of the two genotypes isolated from (D) the AA regime (R^2^ = 0.19, P<0.001) or (F) the non-AA regime (R^2^ = 0.41, P<0.001).

As expected, auxotrophs were unable to grow when no AAs were present in the environment, yet grew when all AAs were supplemented to the growth medium. This held true for all auxotrophs analyzed from both selection regimes (Fig 4A and 4B). However, coculturing auxotrophic genotypes in the absence of AAs together with the prototrophic strain they had coevolved with, resulted in fitness levels of auxotrophs that were statistically indistinguishable to the levels they have reached in monoculture in the presence of AAs (one-way ANOVA followed by a LSD post-hoc test: P<0.05, n = 4 for 6 derived auxotrophic strains (AA regime), n = 4 for 3 derived auxotrophic strains (non-AA regime), Fig 4) indicating that auxotrophs derived amino acids from the cocultured prototrophs. Supplementing these cocultures with additional AAs further increased the fitness of AA-evolved auxotrophs over the levels they reached under unsupplemented conditions (one-way ANOVA followed by a LSD post-hoc test: P<0.05, n = 4 for 6 derived auxotrophic strains, Fig 4A), while the growth of non-AA-evolved auxotrophs did not change upon AA supplementation (one-way ANOVA followed by a LSD post-hoc test: P>0.05, n = for 3 derived auxotrophic strains, Fig 4B). Interestingly, when the focal auxotrophs were cocultured with the evolutionary ancestor and not the coevolved prototrophs, auxotrophs isolated from the AA regime were generally less fit as compared to the situation when the coevolved prototroph was present (one-way ANOVA followed by a LSD post-hoc test: P<0.05, n = 4 for 6 derived auxotrophic strains, Fig 4A). Still, AA supplementation significantly enhanced the fitness of AA-evolved auxotrophs in coculture with the ancestor over the fitness reached in unsupplemented medium (one-way ANOVA followed by a LSD post-hoc test: P<0.05, n = 4 for 6 derived auxotrophic strains, Fig 4A). This pattern is consistent with a coevolutionary change between derived auxotrophs and prototrophs that is absent when the ancestral prototroph is the interaction partner. In contrast, for auxotrophs that evolved in the non-AA regime, it did not make a difference whether the coevolved prototrophs or the evolutionary ancestor was present when the coculture was exposed to unsupplemented minimal medium (one-way ANOVA followed by a LSD post-hoc test: P>0.05, n = 4 for 3 derived auxotrophic strains, Fig 4B). However, when AAs were provided to these cocultures, the auxotrophs reached the highest fitness levels of all experimental conditions analyzed (one-way ANOVA followed by a LSD post-hoc test: P>0.05, n = 4 for 3 derived auxotrophic strains, Fig 4B).

Together, these observations suggest that either the auxotrophs’ ability to derive AAs from the coexisting prototrophs increased over time or, alternatively, prototrophic cells increased their amino acid production levels. In either way, these results demonstrate that evolved auxotrophs utilized the AAs that were available in the growth environment as well as those they could obtain from other, coexisting strains.

### Negative frequency-dependent selection maintains genotypic diversity

Which ecological mechanism maintained the evolved genotypic diversity (i.e. both auxotrophic and prototrophic genotypes) in the evolution experiment? A likely possibility that has been previously identified to be key for maintaining synthetically engineered cross-feeding genotypes that reciprocally exchange essential AAs is negative frequency-dependent selection [30]. To determine whether the same mechanism also stabilized our naturally-evolved genotypes, the ability of derived auxotrophs and prototrophs to invade a population of the respective other strain when rare was determined in the absence and presence of AAs in the environment. Under all conditions tested, the initially rare type (i.e. auxotroph or prototroph, initial ratio: 1:100) was able to invade a resident population of the respective other strain as evidenced by its significantly increased selection coefficients (one-sample t-test: P<0.05, n = 4 for 6 derived auxotrophic or prototrophic strains for the AA regime, Fig 4C and 4E). Even following the fate of both types over 75 bacterial generations revealed for both auxotrophs and prototrophs a pronounced fitness advantage when rare that steeply declined as the population-level proportion of the focal type increased (linear regression, P<0.05, n = 4 for 6 auxotrophs and prototrophs from the AA regime and n = 4 for 3 auxotrophs and prototrophs from the non-AA regime, Fig 4D and 4F). Interestingly, this pattern was independent of whether or not AAs were supplied to the medium. In other words, the above findings corroborated the hypothesis that AA cross-feeding between prototrophic donor cells and auxotrophic recipients is maintained by negative frequency-dependent selection and that the frequency of both types oscillates around a stable equilibrium point.

To determine whether different growth strategies of the two coevolved genotypes could cause the observed negative frequency-dependent selection, monocultures of the AA-evolved genotypes and the evolutionary ancestor were grown in the presence of AAs and their growth kinetic parameters were compared. This experiment revealed that the derived auxotrophic and prototrophic strains grew significantly faster and achieved a higher cell density than the ancestral strain (one-way ANOVA followed by a LSD post hoc test: P<0.05, n = 4 for 6 derived auxotrophic or prototrophic strains and the ancestor, S2A Fig). Moreover, the growth of auxotrophic strains was characterized by a significantly shorter lag phase (one-way ANOVA followed by a LSD post hoc test: P<0.05, n = 4 for 6 derived auxotrophic or prototrophic strains and the ancestor, S2A Fig) and an earlier onset of the stationary phase than was the case for both derived and ancestral prototrophic genotypes (one-way ANOVA followed by a LSD post hoc test: P<0.05, n = 4 for 6 derived auxotrophic or prototrophic strains and the ancestor, S2B Fig), indicating that auxotrophic strains likely utilized environmentally available AAs until this pool was depleted. In contrast, prototrophic genotypes remained much longer in the exponential growth phase than auxotrophs (one-way ANOVA followed by a LSD post hoc test: P<0.05, n = 4 for 6 derived auxotrophic or prototrophic strains and the ancestor, S2C Fig), suggesting that their growth was limited by the carbon source rather than the amount of AAs present in the environment.

Taken together, these observations suggest that due to their inability to autonomously produce certain amino acids, auxotrophs first utilized the pool of environmentally available AAs before they exploited the AAs produced by other, coexisting strains. This bi-phasic growth pattern together with their obligate dependency on coexisting prototrophs likely maintained auxotrophs by negative frequency-dependent selection.

### Mutations in both structural and regulatory genes caused auxotrophic phenotypes

**Fig 5:**
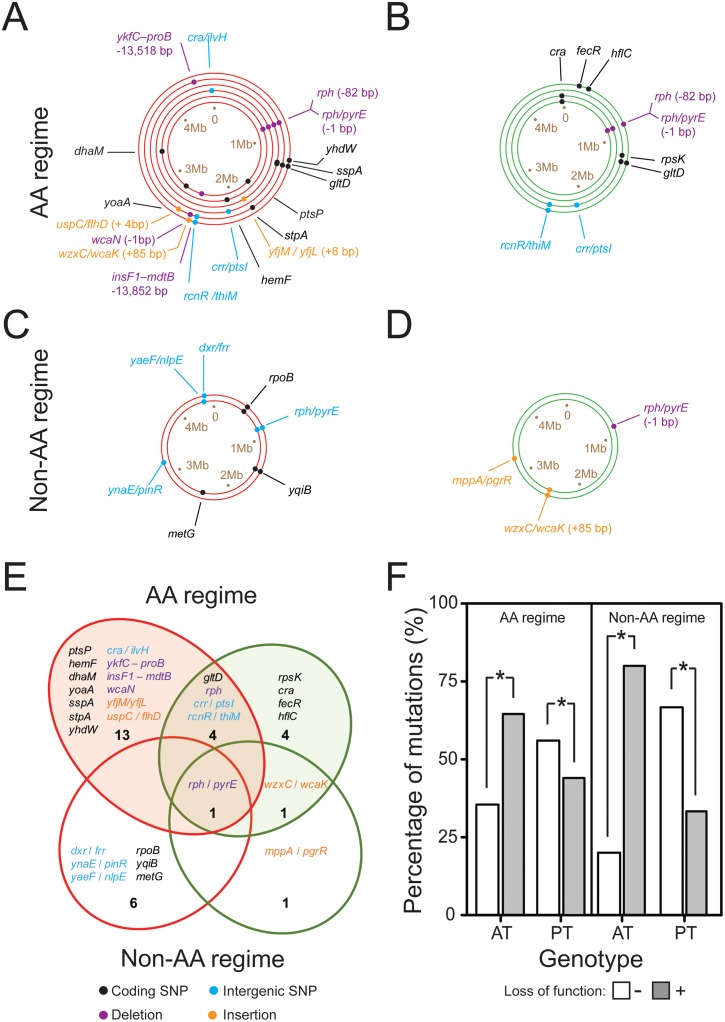
Genomic consequences of adaptation to the two selection regimes. Auxotrophic and prototrophic genotypes that evolved in the two regimes harbor distinct mutations. (A-D) Each ring represents the genome of a single auxotrophic (red rings) or prototrophic (green rings) clone that has been isolated from different replicate populations, which have evolved (A,B) for 2,000 generations under AA-replete, or (C,D) for 1,500 generations under AA-deficient conditions. The order of rings represents the replicate populations from which the cognate auxotroph and prototroph have been isolated. Dots indicate the relative position of the identified mutations within the genome with different colors specifying the type of mutation (see legend). Please see S2 Table for abbreviations of gene names and S3 Table for the identity of genotypes. SNP = single nucleotide polymorphism. (E) Venn diagram summarizing the mutations, which are unique to either auxotrophs (red ellipses) or prototrophs (green ellipses) and those that are shared between both types (overlapping region) for the strains isolated from the AA regime (top, filled ellipses) and non-AA regime (bottom, unfilled ellipses). Numbers indicate the total number of mutations in each sector. Mutations are colored according to their type (see legend). (F) The percentage of mutations present in auxotrophic (AT) or prototrophic (PT) genotypes isolated from the AA regime (left panel) or non-AA regime (right panel) that were predicted to cause a loss (grey bars) or no loss (open bars) in function. Asterisks indicate significant differences in the frequency of both mutation types between auxotrophic and prototrophic genotypes (Pearson’s chi square test: P<0.05, n≥6).

To unravel the genetic basis of the metabolic auxotrophies that emerged in the course of the evolution experiment, genomes of 8 auxotrophs and 6 prototrophs from different replicate populations, which had evolved for up to 2,000 generations in the AA regime (Fig 5A and 5B, 6 auxotrophs and 4 prototrophs, S3 Table) and up to 1,500 generations in non-AA regime (Fig 5C and 5D, 2 auxotrophs and 2 prototrophs, S3 Table), were sequenced and their genome sequence compared to that of the evolutionary ancestor. This analysis revealed that derived auxotrophic mutants carried significantly more mutations in their genome than the coevolved prototrophs (Pearson’s chi square test: P<0.05, n≥6). Moreover, both types had very few mutations in common (Fig 5E, S2 Table), confirming that co-occurring auxotrophic and prototrophic cells represented genetically distinct subpopulations.

Next, we focused on those mutations that arose exclusively in genomes of auxotrophic genotypes with the aim to functionally link them to their auxotrophic phenotypes. Interestingly, auxotrophs isolated from the two selection regimes differed starkly in the mutations present in their genomes (Fig 5E). However, very few of these mutations showed an obvious involvement in AA metabolism. The two auxotrophs isolated from the non-AA regime carried identical SNPs in the *rpoB* gene, which encodes the β-subunit of RNA polymerase (RNAP, b3987, ECK3978). Mutations in this gene are known to result in a specific down-regulation of genes involved in AA biosynthesis [31]. Indeed, both of these genotypes were auxotrophic for the same set of AAs: lysine and tryptophan. In contrast, auxotrophies that arose in the AA-containing environment were caused by completely different mechanisms, including the loss of regulatory and structural genes (Fig 5A and 5E, S2 and S3 Tables). One auxotroph 2-AA-AT (population R2) lost approximately 13 kb from its genome (Fig 5A and 5E, S2 and S3 Tables) due to the deletion of a large fragment that comprised 14 genes. These included the genes encoding the BaeSR two-component regulatory system, loss of which has been previously implicated in the down-regulation of multiple regulatory and AA biosynthesis-associated proteins [32]. Another auxotroph 4-AA-AT (population R5, see S3 Table for description of genotypes) carried a non-sense mutation, which inactivated *sspA*, a gene that encodes the stringent starvation protein A (Fig 5A and 5E, S2 and S3 Tables). This protein is known to be involved in the regulation of AA biosynthesis and mutants lacking this gene have been shown to lose viability under arginine-limiting conditions [33]. The only case, in which a biosynthetic gene was actually lost from the genome was in case of an auxotroph 6-AA-AT that has been isolated from population 6. This mutant had lost a 13 kb region from its genome comprising 20 genes, which included *proA* and *proB*, two genes that are essentially involved in the biosynthesis of the AA proline [34].

However, for most of the observed mutational changes, it remained unclear whether or not a functional link to the cells’ amino acid metabolism existed. To address this issue, we first investigated whether the mutations unique to auxotrophic genotypes inactivated gene functions using the PROVEAN algorithm (S2 Table) [35]. This analysis revealed auxotrophs isolated from both selection regimes bore significantly more loss of function mutations in their genome than the cognate prototrophic types (Fig 5F, Pearson’s chi square test: P<0.05, n≥45).

Next, to determine if the predicted loss-of-function mutations (S2 Table) can negatively affect growth of the corresponding mutants when AA are lacking, each of the 13 mutations that were unique to 6 auxotrophic genotypes (Genotypes: 2-AA-AT, 3-AA-AT, 5-AA-AT, 6-AA-AT, 8-AA-AT, and 8-NA-AT) were transferred into the ancestral background of the prototrophic WT and the growth of the resulting mutants was analyzed in the absence of AAs. Almost half of these reconstructed mutants (46%) did not show detectable growth in the presence of AAs (one sample t-test, P<0.05, n = 8 for each of the 6 auxotrophs tested, S3 Fig), suggesting that these alleles likely caused the observed auxotrophic phenotypes.

In summary, this analysis revealed that genomes of evolved auxotrophic genotypes carried significantly more mutations than their prototrophic counterparts and that mutations identified in auxotrophs were more likely to cause a loss-of-function than the ones detected in prototrophic genotypes.

### The majority of auxotrophy-causing mutations increase bacterial fitness in the presence of amino acids

To determine if auxotrophy-causing mutations increase bacterial fitness in AA-containing environments and were thus selected for, the derived auxotrophic genotypes whose genome sequence has been analyzed (S3 Table) as well as the mutants, in which the identified mutations have been reconstructed (S4 Table), were individually competed against the evolutionary ancestor in the presence of AAs. This included genotypes 2-AA-AT, 3-AA-AT, 5-AA-AT, 6-AA-AT, and 8-AA-AT that evolved in the AA regime, 8-NA-AT that evolved in the non-AA regime, as well as the corresponding 13 mutants that each carried one the reconstructed mutations.

**Fig 6:**
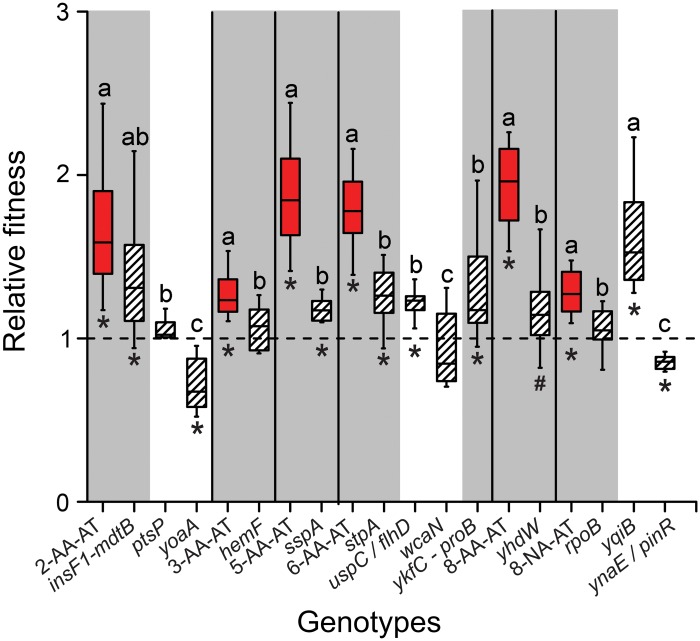
The majority of auxotrophy-causing mutations increase bacterial fitness in the presence of amino acids. A total of 13 mutations, which were unique to auxotrophic genotypes isolated from both the AA (genotypes: 2-AA-AT, 3-AA-AT, 5-AA-AT, 6-AA-AT, 8-AA-AT) and the non-AA regime (genotype: 8-NA-AT), were reconstructed in the ancestral WT background. The phenotypes of the resulting auxotrophic (i.e. grey background) and prototrophic mutants (white background) were determined by quantifying the growth these mutants achieved in unsupplemented minimal medium during 24 h (S3 Fig). Shown are the results of competitive fitness experiments between the ancestral WT and the derived auxotrophs (red boxes; S3 Table) or the reconstructed mutants (hatched boxes; S4 Table) in the presence of AAs. Relative fitness is the ratio of Malthusian parameters and the dashed line represents the fitness of the evolutionary ancestor. Different letters indicate significant fitness differences between the evolved auxotrophs and the reconstructed mutants (one-way ANOVA: P<0.05, n = 8), while symbols below boxes signify the statistical significance levels for the comparison to the evolutionary ancestor (independent sample t-test: * P<0.05, # P = 0.094, n = 8). Box plots: medians (horizontal lines within boxes), interquartile range (boxes), and 1.5x-interquartile range (whiskers).

Interestingly, 4 of the 7 auxotrophy-causing mutations identified (i.e. *insF1- mdtB*, *sspA*, *stpA*, *and ykfC-proB*) significantly increased fitness of the corresponding mutants in the presence of AAs relative to the ancestral strain (independent sample t-test: P<0.05, Fig 6), while one mutant (*yhdW*) showed a trend in this direction (independent sample t-test: P = 0.094, n = 8, Fig 6). Fitness values of the other mutants that carried the three remaining auxotrophy-causing mutations were statistically indistinguishable from the levels of the evolutionary ancestor (independent samples t-test, P<0.08, n = 8 for each of the mutants tested, Fig 6). One of these mutations (in *rpoB*) originated from the auxotroph 8-NA-AT that evolved in the absence of AAs. Interestingly, two of the six mutations that did not cause a metabolic auxotrophy (i.e. *uspC/flhD* and *yqiB*) also gave rise to a fitness advantage in the corresponding mutants when AAs were present in the environment.

Taken together, finding that most of the mutations that resulted in amino acid auxotrophies also increased bacterial fitness in the presence of AAs corroborated that indeed auxotrophy-causing mutations were selectively favored in AA-containing environments.

## Discussion

Why are metabolic auxotrophies so common in natural microbial communities? Hypothesizing that adaptive benefits may account for the frequently observed loss of metabolic functions, our evolution experiment revealed that prototrophic *Escherichia coli* cells rapidly evolved metabolic auxotrophies when adapting to environments that contained all of 20 different AAs. Interestingly, also serial propagation in AA-free environments resulted in the emergence of genotypes that had a lost the ability to autonomously produce some amino acids, yet the number of auxotrophies per strain, the number of auxotrophic strains per population, and the number of populations containing auxotrophs was significantly lower relative to populations that evolved under AA-replete conditions. In line with prior expectations, auxotrophic genotypes that evolved in AA-containing environments gained an adaptive advantage over their evolutionary ancestor, yet the observed fitness benefit was contingent on the presence of AAs in the environment. Surprisingly, evolved auxotrophs also derived amino acids from coexisting prototrophic cells and this interaction was stabilized by negative frequency-dependent selection. Multiple genetic routes lead to the inactivation of AA biosynthetic abilities, including mutations in both regulatory and structural genes. Moreover, reconstructing all mutations identified in auxotrophic genotypes in the ancestral WT background revealed that most auxotrophy-causing mutations that were considered, resulted in an increased fitness of the corresponding mutant in AA-containing environments, thus strongly suggesting that they were selectively favored under the experimental conditions.

A main outcome of the evolution experiment was that adaptive benefits drove the rapid loss of biosynthetic functions when the focal metabolites were sufficiently present in the cell-external environment. These findings are in line with previous analyses, which revealed a significant fitness advantage synthetically engineered, auxotrophic mutants gained over competing prototrophic types when AAs were sufficiently present in the environment [7, 8, 23, 30]. What could explain these adaptive benefits? One explanation could be that the loss of biosynthetic functions in the presence of metabolites in the environment results in energetic savings for a cell that might be due to the saving of protein production costs [7, 8, 23, 36]. Alternatively, a regulatory or metabolic rewiring of cells could provide auxotrophs with a growth advantage in AA-deficient conditions—for example by changing fluxes through the cells’ metabolic networks [37]. Another explanation could be that auxotrophic cells do not only use amino acids as building block metabolites, but also catabolize AAs. This could explain the advantage auxotrophic genotypes gain relative to prototrophic cells, which autonomously produce the AAs they require for growth. Which of these mechanisms causes the fitness increase of auxotrophic genotypes in AA-containing environments, however, remains unknown and should be subject to further investigation.

A prediction that follows from our observations is that metabolic auxotrophies should rapidly evolve whenever bacteria are cultivated in AA-rich media or inhabit environments with increased AA-availabilities [8, 38, 39]. Indeed, metabolic auxotrophies have been repeatedly reported to arise in laboratory-based evolution experiments [11, 40, 41] or have been detected in natural microbial communities [7–10, 26, 30, 42–44]. In our experiment, derived auxotrophs always coexisted together with metabolically autonomous prototrophs. A strikingly similar pattern has been previously observed in populations of *Pseudomonas aeruginosa* that adapted to the lungs of cystic fibrosis (CF) patients: both prototrophic and auxotrophic strains have been isolated from the AA-rich mucus that fills the lungs of these CF patients [44, 45].

Independent of whether or not AAs were present in the selective environment, auxotrophs that evolved in our evolution experiment always obtained AAs also from other community members such as the coexisting prototrophs. Two mechanisms are conceivable how auxotrophs obtained the AAs they required for growth: metabolites might be exchanged among genotypes via diffusion through the cell-external environment [46–48] or, alternatively, in a contact-dependent manner [49, 50]. Recently it has been described that auxotrophic cells of *E*. *coli* can produce so-called ‘nanotubes’ to directly obtain cytoplasmic AAs from other bacterial cells [50]. These structures likely minimize the costs to the AA-producing cell by reducing the loss of AAs to the cell-external environment. Thus, the formation of nanotubes of AA-starved bacteria might be interpreted as a strategy to survive under AA-limiting conditions. Such a scenario could explain the evolution of auxotrophic genotypes in the non-AA regime. A contact-dependent exchange mechanism might also have allowed growth of auxotrophic genotypes after depletion of amino acids in the cell-external environment.

Analyzing the genomes of derived mutants unveiled a diverse spectrum of mutations that caused the observed phenotypes. The finding that auxotrophic genotypes bore on average significantly more loss-of-function mutations than the cognate prototrophs strongly suggests the adaptive loss of functions resulted in the observed auxotrophies (Fig 5D). In contrast to expectations, deactivation of amino acid biosynthetic pathways via a deletion of the corresponding structural genes was much less common than the loss of regulatory elements with putative roles in AA metabolism (Fig 5, S2 Table). Interestingly, auxotrophies that evolved in the non-AA regime were most likely due to mutations that down-regulated the expression levels of AA biosynthetic genes, while most auxotrophies that evolved in the AA-containing environment were caused by a complete loss of enzyme-coding regions or an inactivation of the corresponding regulatory elements. This pattern likely mirrors differences in the two selective regimes. While the environment that did not contain AAs penalized any newly evolved auxotroph, whose metabolic deficiency could not be compensated by any of the prototrophic types present, the AA-replete condition likely permitted many more different auxotrophs to increase in frequency. Indeed, the only auxotrophs that could be detected in the lines that evolved under AA-free conditions had lost the ability to produce leucine, lysine, and tryptophan, which incur relatively low metabolic costs [51] and are thus cheaper for the corresponding prototrophs to produce. In contrast, in the AA-replete environment, many more auxotrophic mutants evolved, with all replicate populations displaying a core set of common auxotrophies (S1 Fig). Since both epistatic interactions among mutations [7] and metabolic costs to produce the corresponding amino acids [8] determine the fitness consequences of a biosynthetic loss-of-function mutation in *E*. *coli*, the observation of such strikingly parallel changes likely reflects selective constraints acting on the evolved populations.

Fitness consequences resulting from the individually reconstructed, auxotrophy-causing mutations were in a majority of cases different from the fitness levels the derived auxotrophic strain achieved (Fig 6). Given that all evolved genomes analyzed contained multiple mutations, the fact that one individual mutation could not in all cases explain the fitness of the whole organism likely resulted from epistatic interactions among mutations that have been previously shown to strongly affect the fitness of auxotrophic genotypes [7]. This means that those auxotrophy-causing mutations that did not cause an increased fitness in the reconstructed mutant may have been adaptive in the genetic background in which the mutation arose. In addition, also an accumulation of multiple beneficial mutations in the same genetic background could explain why reconstructing the auxotrophy-causing mutation in the ancestral background was not in all cases sufficient to reconstitute the fitness level of the derived genotype, from which this mutation originated. Unfortunately, due to a lack of clear evolutionary lineages, these hypotheses escape an experimental validation. Interestingly, some of the mutations that did not cause an AA auxotrophy were found to be neutral (1 case) or even deleterious (2 cases) (Fig 6). These mutations likely hitchhiked on another adaptive mutation that arose in the focal genotype–a phenomenon that has been observed previously in other selection experiments [25, 52].

Theoretical predictions of metabolic auxotrophies in otherwise uncharacterized bacterial genomes have been largely based on whether or not a given biosynthetic pathway exists in the focal organism [7–9]. Due to a lack of understanding of the underlying regulatory networks, these approaches usually neglect the multifarious genetic routes that can possibly cause metabolic auxotrophies. Consequently, previously published estimates that only consider the absence or presence of biosynthetic genes [7–9] likely underestimate the true number of auxotrophic prokaryotes in nature dramatically. Given that the fitness advantage multiply auxotrophic bacteria gain are strongly affected by epistatic interactions among the auxotrophy-causing mutations [7], mutationally-induced regulatory changes could represent an effective bypass of this evolutionary constraint.

The adaptive loss of metabolic capabilities and the emergent dependence on other co-occurring strains as observed in this study have significant ramifications for the evolution of bacterial genomes. A striking pattern that emerges when genomes of multiple different bacterial clones are sequenced that have coexisted together for extended time periods, is not only the frequent loss of many biosynthetic functions from their genomes, but often also a high degree of metabolic complementarity on the genomic level. Examples involve both free-living bacterial communities [53] and consortia of endosymbiotic bacteria, whose metabolite production is intricately interwoven between their eukaryotic host [12, 54, 55] and other coevolving bacteria [14, 56, 57]. In the latter case, loss of biosynthetic functions has been suggested to arise as a consequence of drift resulting from periodic bottlenecks leading to low population sizes. Empirical evidence, however, suggests that the bottleneck size experienced by bacterial symbionts during transfer between insect hosts is usually around 10^3^ CFUs [58, 59]. This population size is strikingly similar to the number of bacterial cells that were serially passaged in our evolution experiment. Assuming the cytoplasm of host cells is as nutrient-rich as the medium used in this study, the evolution of metabolic complementarities in insect endosymbionts could be selectively favored as well.

Given the ease, with which metabolic auxotrophies evolve, thereby rendering the resulting mutants dependent on other coexisting organisms, it is conceivable how this event can set the stage for a coevolutionary race, in which the interacting partners may further benefit from losing additional metabolic functions. This race will most likely favor those loss-of-function mutants, which are fitter than other competing genotypes given the presence of a donor that can sufficiently compensate for their deficiencies. This ‘black-queen’-like process [46, 48] can then lead to coadaptations on both sides. Indeed, our observation that the AA-evolved auxotrophs grew significantly better when cocultured with the derived prototrophs than their evolutionary ancestor supports this interpretation (Fig 4A). In the long-run, this process should lead to metabolic networks that are intricately interconnected between multiple different bacterial genotypes. Ultimately, the findings of our study may provide a plausible explanation of why most bacterial species known are difficult to cultivate under laboratory conditions [60, 61]: they have most likely adapted to the nutritional biotic and abiotic environment they encountered in nature, which complicates a reproduction of these conditions in the laboratory.

## Material and Methods

### Strains, media, and growth conditions

The isogenic ancestor of the evolution experiment was *Escherichia coli* BW25113 Ara^-^ or Ara^+^. The Ara^+/-^ phenotypic marker (i.e. presence/ absence of the *araA* gene [7, 8]) was used to phenotypically discriminate strains (S3 Table) (e.g. ancestral and derived strain) by red-white differentiation. When growing on tryptone agar (TA) containing 10% arabinose and 0.5% tri-phenyl tetrazolium chloride (TTC) [62], Ara^-^ colonies release basic end products, while Ara^+^ colonies release acidic end products. This causes a color change of the pH-dependent TTC dye [62], giving rise to white (Ara^-^) and red colonies (Ara^+^).

Unless stated otherwise, liquid minimal medium for *Azospirillum brasillense* (MMAB) [63] with 0.5% fructose as carbon source and without biotin was used for cultivating bacteria in all experiments. For some experiments, all 20 AAs (+AA regime) were supplemented to the MMAB medium—each at a concentration of 100 μM. When solid MMAB medium was used, 1.5% agar was supplemented to the liquid minimal medium. For all experimental assays, strains and populations were first precultured in the conditions they evolved in before the experimental assay was inoculated (1:100 dilution). All precultures and growth assays were performed at 30°C with shaking at 220 rpm for 18 or 24 hours, respectively. All experiments involving monocultures were initiated using ~10^5^ cells ml^-1^ of the focal strain or population and cocultures were inoculated with ~10^5^ cells ml^-1^ of each strain or population.

### Evolution experiment

Eight independent lineages were founded from four isogenic clones of either *E*. *coli* BW25113 (Ara^-^) [64] or *E*. *coli* BW25113 (Ara^+^) [8]. The eight lineages were serially propagated for 2,000 generations by daily transfers into 1 ml of fresh MMAB medium containing a mixture of all 20 amino acids (i.e. AA regime) or 1 ml of unsupplemented MMAB medium (i.e. non-AA regime). Each day, 1 μl of culture medium was transferred to 999 μl of fresh medium (1:1,000 dilution). The transfer volume contained ~10^3^ cells and the population expanded to ~10^6^ cells after 24 hours of growth, resulting in ~10 generations ever day (generations = [(log number of cells after 24 h)—(log number of cells initially present)]/ 0.301). To ensure the size of the population bottleneck was consistent during the course of the experiment, the number of colony forming units (CFUs) was determined in regular intervals (i.e. every 150 generations) and the dilutions adjusted accordingly. Evolving populations were frozen at -80°C at 250, 500, 1,000, 1,500, and 2,000 generations along with 50% glycerol for subsequent experiments.

### Measurement of growth parameters

Productivity of the evolving populations was measured by determining the number of CFUs ml^-1^ on Lysogeny Broth (LB) agar plates at 15, 180, 450, 750, 870, 1,170, 1,365, 1,590, 1,695, 1,920, and 1,995 generations. Growth kinetic parameters like the maximal growth rate (μ_max_ h^-1^) and the maximum optical density at 600 nm reached during 24 h (OD_max_) were determined for populations and clones isolated from different time points over the course of the evolution experiment. For this, frozen samples were revived by inoculating 10 μl into 1 ml of the corresponding medium as described above. Growth assays were performed in 50 μl of medium in a 384 micro-well plate (Greiner, Germany) and growth kinetics were monitored in a Tecan Infinite Pro Microplate reader (Tecan, Austria) by recording the OD every eight minutes for 24 h at 30°C with shaking at 2.5 Hz in the interim. Calculating the percent change between the maximal growth rates that a genotype achieved during 24 h of growth in the presence of AAs (20 AAs each at a concentration of 100 μM, μ_max_ h^-1^_+AA_) from the values reached under AA-free conditions (μ_max_ h^-1^_-AA_) provided a quantitative measure to compare the growth of a specific population under both conditions. This was calculated using the formula:

$$\text{Percent change }=\;\left\{[(\mu_{\text{max}}\;{\text{h}}^{-1}{}_{-\text{AA}}-\mu_{\text{max}}\;{\text{h}}^{-1}{}_{+\text{AA}})\text{ x }100\;]/\mu_{\text{max}}\;{\text{h}}^{-1}{}_{+\text{AA}}\right\}\;-100$$

Positive values indicate the population growths better in the absence of AAs, while negative values point to a growth-stimulating effect of AAs. This experiment has been performed with the ancestral as well as the eight derived populations after 250, 500, 1,000, 1,500, and 2,000 generations of evolution in the absence and presence of AAs. Each comparison has been replicated 4 times.

### Determination of auxotrophies

The emergence of auxotrophic mutants in the evolving populations was determined by resuscitating and preculturing frozen samples from generations 250, 500, 1,000, 1,500, and 2,000 of both selection regimes. These cultures were then serially diluted such that each population contained ~10^3^ CFUs ml^-1^ and plated on MMAB agar plates that contained all AAs. 1,000 colonies from each population of the two regimes were then inoculated onto a fresh MMAB agar plate that contained all AAs. The colonies were then replicated on MMAB agar plates without any AAs using a 96-pin replicator to identify colonies that were unable to grow. Any colony that failed to grow on MMAB without AAs was deemed auxotrophic. The colonies identified in this way were then replica-plated on MMAB plates without AAs and 20 different MMAB ‘drop-out’ media—each containing a different combination of 19 AAs, leaving out one specific AA [65]. This approach allowed determining specific AA-auxotrophies of the focal strains. Strains that were unable to grow on MMAB without AA supplementation yet could grow on all 20 drop-out media, were deemed ‘non-assigned’ (NA).

### Competitive fitness assays

The fitness of each auxotrophic or prototrophic type that has been isolated after 1,000, 1,500, and 2,000 generations of growth in the AA regime and after 500 and 1,500 generations in the non-AA regime was determined in competition experiments against the evolutionary ancestor. For this, ~10^5^ cells of the derived clone as well as of the evolutionary ancestor that was carrying the respective other Arabinose utilization marker (i.e. Ara^+^ versus Ara^-^ and *vice versa*) were precultured and subsequently co-inoculated into 1 ml of MMAB with or without AAs. The relative fitness of strains from the AA regime was determined in the absence or presence of AAs, while the strains from the non-AA regime were only analyzed in the absence of AAs. The number of CFUs ml^-1^ was determined at the onset (i.e. 0 h) and at the end of the coculture period (i.e. after 24 h) by plating on TA agar plates and the Malthusian fitness parameter was calculated as described previously [62]. Relative fitness measurements of each individually isolated genotype were replicated 8 times. A similar approach was used to determine the fitness effects of each of the 13 mutations that have been transferred to the WT background. The reconstructed strains that each carried a single mutation (Ara^-^) were competed against the evolutionary ancestor (Ara^+^) in MMAB with AAs. The relative fitness of this comparison was determined as described above.

### Metabolic dependency

To determine to which extent the growth of derived auxotrophs depended on the availability of AAs in the environment and/ or the presence of a cocultured prototroph, six auxotrophic genotypes that have been isolated after 2,000 generations of evolution in the AA regime or two isolated after 500 and 1,500 generations from the non-AA regime were precultured in AA-supplemented MMAB medium. The evolutionary ancestor or the corresponding prototrophs, which have been isolated from the same population and the same time points as the auxotroph, were similarly precultured in MMAB medium with or without AA-supplementation, depending on the medium of the main experiment. ~10^5^ CFUs of the focal auxotrophic mutant were inoculated into 1 ml MMAB with or without AAs. The same experimental set-up was repeated three times: (i) with the auxotrophs grown in monoculture, or by co-inoculating ~10^5^ CFUs per ml of (ii) a coevolved prototrophic strain, or (iii) the evolutionary ancestor. Each experimental treatment was replicated 4-times. All of these cultures were incubated for 24 h. The number of CFUs ml^-1^ was determined for each strain at the start (0 h) and the end of the experiment (24 h). Since the derived auxotrophic and prototrophic types in each coculture carried the same Ara marker (because they descend from the same ancestor), both types were distinguished by plating on an unsupplemented MMAB plate and LB plates. In this way, the number of CFUs ml^-1^ of prototrophic or ancestral strains (i.e. CFUs on the MMAB plate) and CFUs ml^-1^ of the auxotroph (i.e. CFUs on the LB plate minus CFUs on the MMAB plate) were determined and the Malthusian parameter of the auxotrophic strain calculated as described [62].

### Invasion-from-rare experiment

To determine the frequency-dependence of the interaction between the evolving auxotrophic and prototrophic genotypes, cultures of auxotrophs and the corresponding prototrophs that have been isolated after 2,000 generations of evolution in the AA regime (n = 6) or after 500 (n = 1) and 1,500 generations (n = 2) of growth in the non-AA regime were precultured. Subsequently, cocultures between both partners were inoculated together at different initial frequencies (i.e. 1:100 or 100:1), such that the total initial cell density of the coculture was 10^5^ CFUs ml^-1^. Each experimental treatment was replicated 4-times and cocultures were propagated for 75 generations. This was done by transferring 1 μl of culture medium to 999 μl of fresh medium (1:1,000 dilution) every 24 h, resulting in a transfer of ~10^3^ CFUs (i.e. 15 bacterial generations) per day, akin to the conditions of the evolution experiment. The prototrophic or ancestral strains that derived from the same population were plated and distinguished as described above at the onset and after every transfer cycle. The number of CFUs ml^-1^ was determined for both types and the selection coefficients of the invading (rare) type at the end of every transfer cycle was calculated as described [66].

### Whole-genome re-sequencing

To understand the genomic basis of the observed phenotypic auxotrophies in the evolving populations, representative auxotrophic and prototrophic strains from both selection regimes were selected for genome sequencing. From the AA-supplemented environment, six auxotrophs and four coevolved prototrophs were selected from generation 2,000. In addition, two auxotrophs from generation 1,500 and two coevolved prototrophic isolates from the unsupplemented regime were also selected for sequencing. Genomic DNA was extracted after these strains have been grown in LB medium for 24 h using the Epicentre MasterPure DNA extraction kit (Biozym Scientific, Germany). Quality control and library preparation (TruSeq, Illumina) was performed by the Max Planck Genome Centre Cologne, Germany (http://mpgc.mpipz.mpg.de/home/) and sequencing was performed on the Ilumina HiSeq2500 platform. The resulting raw Illumina sequences were aligned to the published reference genome of *Escherichia coli* BW25113 (CP009273_1) [67] using the *breseq* pipeline and mutations were thus identified [68, 69]. The effect of single nucleotide polymorphisms (SNPs) on gene function was determined using the PROVEAN algorithm [35], which predicts whether a given SNP has a neutral or deleterious effect on the protein activity of the gene product it encodes. Whole gene deletions were by default classified as being deleterious for gene function.

### Reconstruction of single mutations in the WT strain

The 13 mutations that were unique to 6 evolved auxotrophs (2-AA-AT, 3-AA-AT, 5-AA-AT, 6-AA-AT, 8-AA-AT, and 8-NA-AT; see S3 Table) were individually moved into the *E*. *coli* BW25113 strain using the Scarless Cas9 Assisted Recombineering (no-SCAR)-system described previously [70]. The plasmids pCas9cr4 (accession number: 62655) and pKDsgRNA-ack (accession number 62654) for the protocol were obtained from Addgene. Briefly, the WT BW 25113 was sequentially transformed with the pCas9cr4 plasmid and then with the plasmid containing the guide RNA (sgRNA) pKDsgRNA-X, where X is specific sequence of the sgRNA targeting the genomic region to be mutated. Protospacer targeting sequences of the sgRNA (20 bp) were designed using the CRISPR-ERA online tool (http://crispr-era.stanford.edu/). Strains bearing the pCas9cr4 and pKD-sgRNA-X plasmids were then transformed with oligonucleotides containing the mutation of interest (obtained from Integrated DNA Technologies, Germany) and recombination initiated by inducing λ-Red recombinase and Cas9. Transformants were cured of the cloning plasmids by incubating the colonies at 37°C. Subsequently, the focal genomic region of the resulting strains was amplified by PCR and Sanger-sequenced to verify whether they carried the mutation of interest.

### Statistical analysis

Normal distribution of data was assessed using the Kolmogorov-Smirnov test. Homogeneity of variances was determined by applying Levene’s test and variances were considered to be homogeneous when P>0.05. Independent sample t-tests were employed to compare fitness and growth rates of populations evolved under the two regimes, fitness of auxotrophic or prototrophic strains relative to the evolutionary ancestor, and optical density of the reconstructed mutants relative to uninoculated medium. Paired samples t-tests were used to compare growth rates of AA-evolved populations in the presence or absence of amino acids. The cell density of replicate populations over evolutionary time was fitted by exponential fits and the slopes of the fitted lines were compared by employing independent sample t-tests [71–73]. One-way ANOVAs followed by LSD post-hoc tests were used to statistically compare Malthusian parameters in the metabolic dependence experiments as well as the competitive fitness of reconstructed mutants and evolved auxotrophs. The statistical relationship between initial frequencies of the two genotypes and the selection coefficients of the initially rare genotype was tested using linear regression analysis. One sample t-tests determined if selection coefficients of the auxotrophic genotypes were significantly different from 0. P-values were corrected for multiple testing by applying the false discovery rate (FDR) procedure of Benjamini *et al*. [74].

## Supporting Information

S1 Fig Cumulative distribution of amino acid auxotrophies in all evolved auxotrophs. Shown is the relative frequency with which certain auxotrophies have been detected in all auxotrophic genotypes isolated from (A) the AA regime and (B) the non-AA regime. NA indicates cases that could not be attributed to a specific amino acid auxotrophy.
(PDF)

S2 Fig Growth kinetic parameters of the ancestor as well as of cognate pairs of auxotrophic and prototrophic strains that evolved in the AA-regime. (A) Maximum growth rate (μ_max_ h^-1^), (B) duration of the lag phase (h), and (C) duration of the growth phase (h) of the evolutionary ancestor (Anc) as well as the derived auxotrophic (AT) and prototrophic (PT) strains that have been isolated from the AA regime. Different letters above boxes denote significant differences between genotypes (one-way ANOVA followed by a LSD post hoc test: P<0.05, n = 4). Boxplots: median (horizontal lines within boxes), interquartile range (boxes), and 1.5x- interquartile range (whiskers)
(PDF)

S3 Fig A subset of mutations cause auxotrophy in evolved genotypes. Shown is the maximum optical density (OD) the ancestral genotype (green bar), evolved auxotrophs (red bars), and reconstructed mutants (hatched bars) reached over the course of 24 hours of growth in unsupplemented minimal medium. Asterisks indicate significant differences (one sample t-test: P<0.05, n = 8) from the OD of uninoculated medium after 24 h (i.e. no growth). For a description of mutations and evolved strains see S2, S3 and S4 Tables.
(PDF)

S1 Table Evolved strains used for the different experiments. For experiments in Figs 3, 4 and 5, a subset of the isolated auxotrophic and prototrophic strains was chosen, which represented different auxotrophic profiles, thus enabling an analysis of diverse auxotrophic phenotypes that evolved during the course of the experiment. Furthermore, in case of the AA-regime for Fig 4A, 4C and 4D, only a subset of strains isolated from the populations at the end of the evolution experiment (i.e. after 2,000 generations) was used. In all experiments comparing auxotrophic and prototrophic strains, always pairs of cognate genotypes have been used that had been isolated from the same population. Prototrophic strains that were sequenced (S3 Table) were also included in experiments for Figs 3 and 4. Strain type: AT = auxotrophic, PT = prototrophic. *Regime* refers to the selection regime, from which mutants have been isolated: AA = presence of amino acids, non-AA = absence of amino acids. *Replicates* indicates the number of independent replicates used for each of the different strains.
(PDF)

S2 Table Non-synonymous mutations identified in the genomes of derived genotypes and their predicted impact on protein functioning. The effect of mutations in coding regions on the functioning of the corresponding protein was predicted using the PROVEAN algorithm [35]. Gene names and their functional assignment are based on Ecocyc [75]. *Strain identity* code: X-Y-Z where X refers to a particular population (1–8), Y refers to the regime, i.e. AA = amino acid, or NA = no amino acid, and Z refers to the phenotype, i.e. either AT = auxotrophic or PT = prototrophic. *Type of change* indicates whether the mutation was identified in a structural (**S**, encoding functions directly involved in enzymatic reactions of biosynthesis pathways) or a regulatory gene (**R**, encoding functions, which can directly or indirectly control expression or activity of enzymes involved in amino acid biosynthesis). Genes and proteins with unknown functional annotations are designated as ‘NA’. Mutations in structural genes: AT: 18, PT: 5, mutations in regulatory genes: AT: 19, PT: 6.
(PDF)

S3 Table Genotypic and phenotypic characteristics of sequenced auxotrophic and prototrophic genotypes. The genomic features are also illustrated in Fig 5. *Strain identity* code: X-Y-Z where X refers to a particular population (1–8), Y refers to the regime, i.e. AA = amino acid, or NA = no amino acid, and Z refers to the phenotype, i.e. either AT = auxotrophic or PT = prototrophic.
(PDF)

S4 Table Strains used in this study. Abbreviations: Ara^+/-^ = ability to use arabinose as a carbon source present/ absent, WT = wild type.
(PDF)
