# Supplementary material for: Experimental Evolution of Metabolic Dependency in Bacteria
Source: PLoS Genet. 2016 Nov 4;12(11):e1006364. doi: 10.1371/journal.pgen.1006364 (PMC5096674; doi:10.1371/journal.pgen.1006364)
Supplement: S1 Fig — Shown is the relative frequency with which certain auxotrophies have been detected in all auxotrophic genotypes isolated from (A) the AA regime and (B) the non-AA regime. NA indicates cases that could not be attributed to a specific amino acid auxotrophy. (PDF) [file pgen.1006364.s001.pdf]

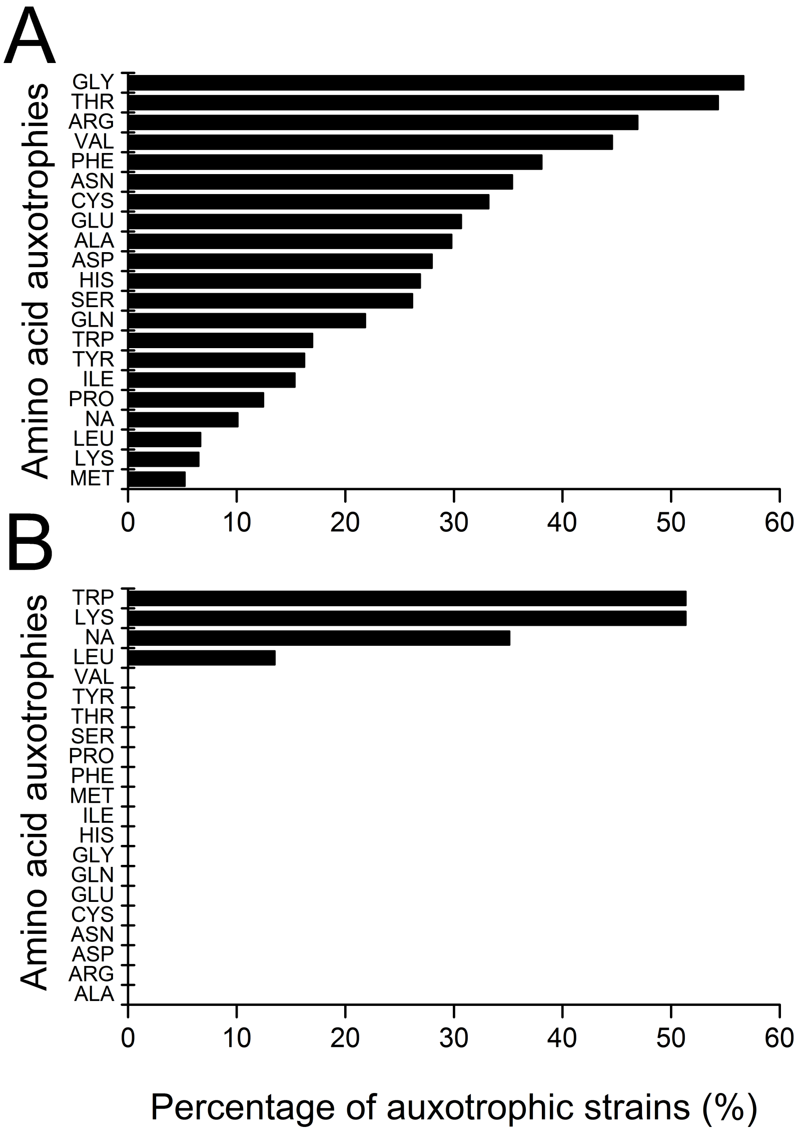

S1 Fig. Cumulative distribution of amino acid auxotrophies in all evolved auxotrophs. Shown is the relative frequency with which certain auxotrophies have been detected in all auxotrophic genotypes isolated from (A) the AA regime and (B) the non-AA regime. NA indicates cases that could not be attributed to a specific amino acid auxotrophy.
