# Supplementary material for: Experimental Evolution of Metabolic Dependency in Bacteria
Source: PLoS Genet. 2016 Nov 4;12(11):e1006364. doi: 10.1371/journal.pgen.1006364 (PMC5096674; doi:10.1371/journal.pgen.1006364)
Supplement: S2 Fig — (A) Maximum growth rate (μmax h-1), (B) duration of the lag phase (h), and (C) duration of the growth phase (h) of the evolutionary ancestor (Anc) as well as the derived auxotrophic (AT) and prototrophic (PT) strains that have been isolated from the AA regime. Different letters above boxes denote significant differences between genotypes (one-way ANOVA followed by a LSD post hoc test: P<0.05, n = 4). Boxplots: median (horizontal lines within boxes), interquartile range (boxes), and 1.5x- interquartile range (whiskers) (PDF) [file pgen.1006364.s002.pdf]

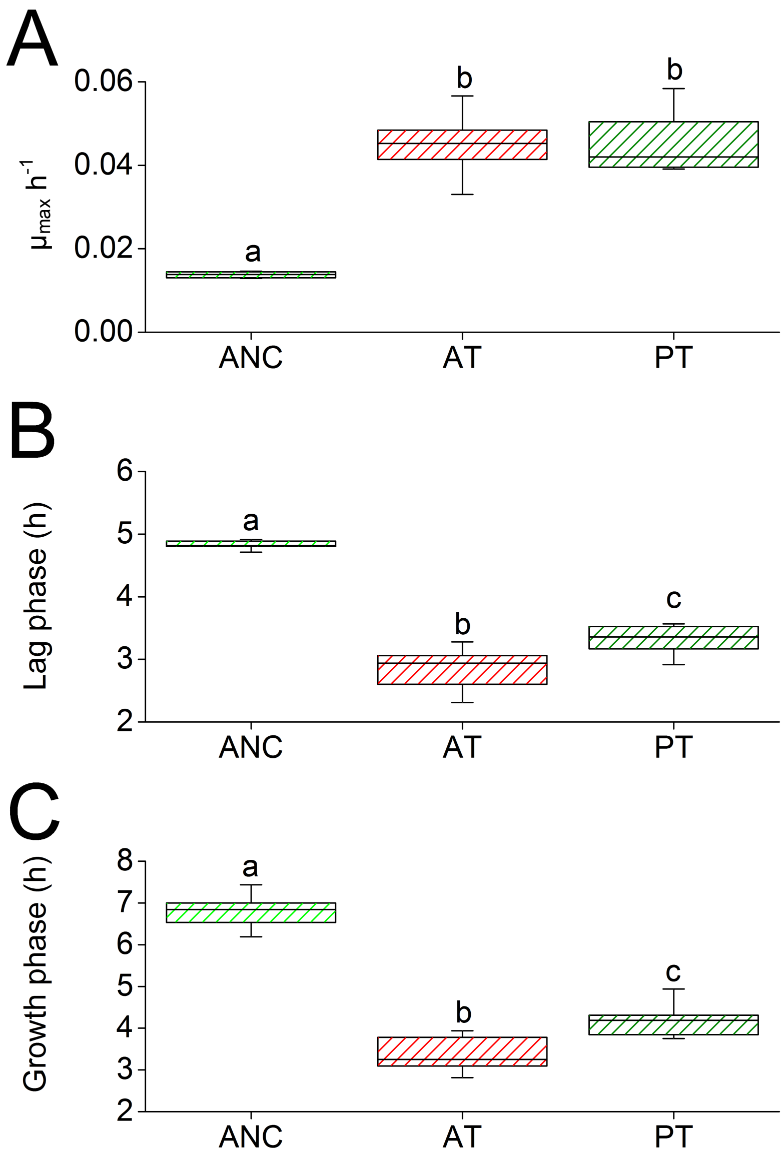

S2 Fig. Growth kinetic parameters of the ancestor as well as of cognate pairs of auxotrophic and prototrophic strains that evolved in the AA-regime. (A) Maximum growth rate (μ_{max} h^{-1}), (B) duration of the lag phase (h), and (C) duration of the growth phase (h) of the evolutionary ancestor (Anc) as well as the derived auxotrophic (AT) and prototrophic (PT) strains that have been isolated from the AA regime. Different letters above boxes denote significant differences between genotypes (one-way ANOVA followed by a LSD post hoc test: $P<0.05$, $n=4$). Boxplots: median (horizontal lines within boxes), interquartile range (boxes), and 1.5x-interquartile range (whiskers)
