# Supplementary material for: Experimental Evolution of Metabolic Dependency in Bacteria
Source: PLoS Genet. 2016 Nov 4;12(11):e1006364. doi: 10.1371/journal.pgen.1006364 (PMC5096674; doi:10.1371/journal.pgen.1006364)
Supplement: S3 Fig — Shown is the maximum optical density (OD) the ancestral genotype (green bar), evolved auxotrophs (red bars), and reconstructed mutants (hatched bars) reached over the course of 24 hours of growth in unsupplemented minimal medium. Asterisks indicate significant differences (one sample t-test: P<0.05, n = 8) from the OD of uninoculated medium after 24 h (i.e. no growth). For a description of mutations and evolved strains see S2, S3 and S4 Tables. (PDF) [file pgen.1006364.s003.pdf]

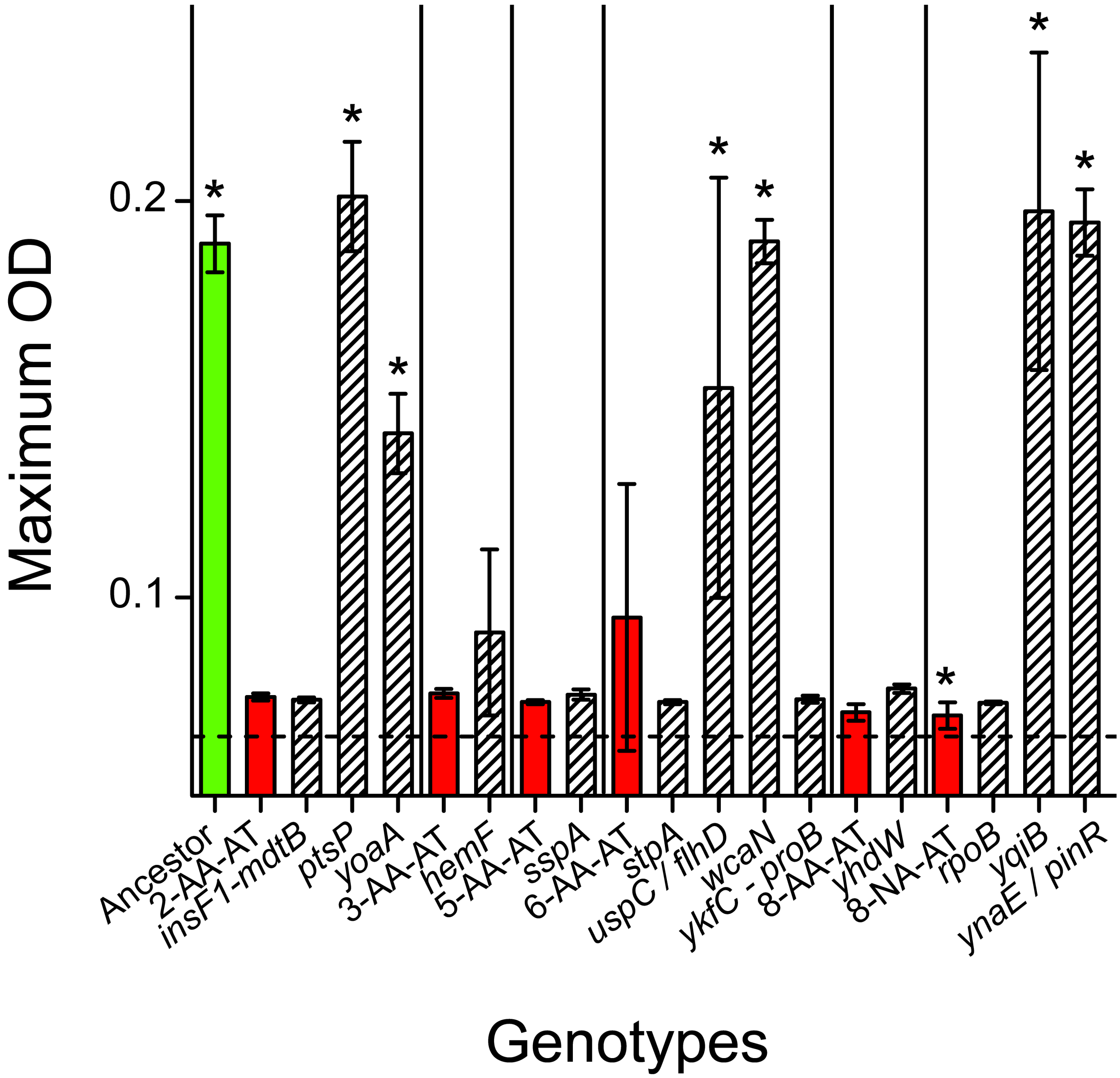


**S3 Fig. A subset of mutations cause auxotrophy in evolved genotypes.** Shown is the maximum optical density (OD) the ancestral genotype (green bar), evolved auxotrophs (red bars), and reconstructed mutants (hatched bars) reached over the course of 24 hours of growth in unsupplemented minimal medium. Asterisks indicate significant differences (one sample t-test: $P<0.05$, $n=8$) from the OD of uninoculated medium after 24 h (i.e. no growth). For a description of mutations and evolved strains see S2-, S3- and S4 tables.
