# Supplementary material for: Experimental Evolution of Metabolic Dependency in Bacteria
Source: PLoS Genet. 2016 Nov 4;12(11):e1006364. doi: 10.1371/journal.pgen.1006364 (PMC5096674; doi:10.1371/journal.pgen.1006364)
Supplement: S1 Table — For experiments in Figs 3, 4 and 5, a subset of the isolated auxotrophic and prototrophic strains was chosen, which represented different auxotrophic profiles, thus enabling an analysis of diverse auxotrophic phenotypes that evolved during the course of the experiment. Furthermore, in case of the AA-regime for Fig 4A, 4C and 4D, only a subset of strains isolated from the populations at the end of the evolution experiment (i.e. after 2,000 generations) was used. In all experiments comparing auxotrophic and prototrophic strains, always pairs of cognate genotypes have been used that had been isolated from the same population. Prototrophic strains that were sequenced (S3 Table) were also included in experiments for Figs 3 and 4. Strain type: AT = auxotrophic, PT = prototrophic. Regime refers to the selection regime, from which mutants have been isolated: AA = presence of amino acids, non-AA = absence of amino acids. Replicates indicates the number of independent replicates used for each of the different strains. (PDF) [file pgen.1006364.s004.pdf]

S1 Table. Evolved strains used for the different experiments.

For experiments in Figs. 3, 4 and 5, a subset of the isolated auxotrophic and prototrophic strains was chosen, which represented different auxotrophic profiles, thus enabling an analysis of diverse auxotrophic phenotypes that evolved during the course of the experiment. Furthermore, in case of the AA-regime for Fig. 4 A, C and D, only a subset of strains isolated from the populations at the end of the evolution experiment (i.e. after 2,000 generations) was used. In all experiments comparing auxotrophic and prototrophic strains, always pairs of cognate genotypes have been used that had been isolated from the same population. Prototrophic strains that were sequenced (table S3) were also included in experiments for Figs. 3 and 4. Strain type: AT = auxotrophic, PT = prototrophic. *Regime* refers to the selection regime, from which mutants have been isolated: AA = presence of amino acids, non-AA = absence of amino acids. *Replicates* indicates the number of independent replicates used for each of the different strains.

Experiment (Figure)	Strain type	Generation/s; Regime	Number of strains used	Replicates
**Distribution of auxotrophies (Fig 2A, C and S1A)**	AT	1,000, AA	60	1
	AT	1,500, AA	256	1
	AT	2,000, AA	157	1
**Distribution of auxotrophies (Fig 2B, D and S1B)**	AT	500, Non-AA	18	1
		1,500, Non-AA	19	1
**Fitness relative to ancestor (Fig 3A)**	AT	1,000, AA	8	4
	AT	1,500, AA	5	4
	AT	2,000, AA	15	4
	PT	1,000, AA	8	4
	PT	1,500, AA	5	4
	PT	2,000, AA	15	4
**Fitness relative to ancestor (Fig 3B)**	AT	500, Non-AA	1	4
	AT	1,500, Non-AA	2	4
	PT	500, Non-AA	1	4
	PT	1,500, Non-AA	2	4
**Dependency on prototroph/ancestor (Fig 4A)**	AT	2,000, AA	6 (Table S3)	4
	PT	2,000, AA	6 (4 from Table S3)	4
**Dependency on prototroph/ancestor (Fig 4B)**	AT	500, Non-AA	1	4
	AT	1,500, Non-AA	2 (Table S3)	4
	PT	500, Non-AA	1	4
	PT	1,500, Non-AA	2 (Table S3)	4
**Invasion from rare (Fig 4C,E)**	AT	2,000, AA	6 (Table S3)	4
	PT	2,000, AA	6 (4 from Table S3)	4
**Invasion-from-rare (Fig 4D,F)**	AT	500, Non-AA	1	4
	AT	1,500, Non-AA	2 (Table S3)	4
	PT	500, Non-AA	1	4
	PT	1,500, Non-AA	2 (Table S3)	4
**Growth kinetics (Fig S2)**	AT	2,000, AA	6 (Table S3)	4
	PT	2,000, AA	6 (4 from Table S3)	4
