# Supplementary material for: Experimental Evolution of Metabolic Dependency in Bacteria
Source: PLoS Genet. 2016 Nov 4;12(11):e1006364. doi: 10.1371/journal.pgen.1006364 (PMC5096674; doi:10.1371/journal.pgen.1006364)
Supplement: S2 Table — The effect of mutations in coding regions on the functioning of the corresponding protein was predicted using the PROVEAN algorithm [35]. Gene names and their functional assignment are based on Ecocyc [75]. Strain identity code: X-Y-Z where X refers to a particular population (1–8), Y refers to the regime, i.e. AA = amino acid, or NA = no amino acid, and Z refers to the phenotype, i.e. either AT = auxotrophic or PT = prototrophic. Type of change indicates whether the mutation was identified in a structural (S, encoding functions directly involved in enzymatic reactions of biosynthesis pathways) or a regulatory gene (R, encoding functions, which can directly or indirectly control expression or activity of enzymes involved in amino acid biosynthesis). Genes and proteins with unknown functional annotations are designated as ‘NA’. Mutations in structural genes: AT: 18, PT: 5, mutations in regulatory genes: AT: 19, PT: 6. (PDF) [file pgen.1006364.s005.pdf]

S2 Table. Non-synonymous mutations identified in the genomes of derived genotypes and their predicted impact on protein functioning. The effect of mutations in coding regions on the functioning of the corresponding protein was predicted using the PROVEAN algorithm [1]. Gene names and their functional assignment are based on Ecocyc [2]. *Strain identity* code: X-Y-Z where X refers to a particular population (1-8), Y refers to the regime, i.e. AA = amino acid, or NA = no amino acid, and Z refers to the phenotype, i.e. either AT = auxotrophic or PT = prototrophic. *Type of change* indicates whether the mutation was identified in a structural (**S**, encoding functions directly involved in enzymatic reactions of biosynthesis pathways) or a regulatory gene (**R**, encoding functions, which can directly or indirectly control expression or activity of enzymes involved in amino acid biosynthesis). Genes and proteins with unknown functional annotations are designated as 'NA'. Mutations in structural genes: AT: 18, PT: 5, mutations in regulatory genes: AT: 19, PT: 6.

Gene, Blattner ID, accession number	Strain identity	Protein function	Mutation	Predicted effect	Type of change
ptsP, b2829, ECK2825	2-AA-AT	Phosphoenol-pyruvate-protein phosphor-transferase	M604R	Neutral	S
yoaA, b1808, ECK1806	2-AA-AT	Conserved protein	D121Y	Neutral	NA
hemF, b2436, ECK2431	3-AA-AT	Coproporphyrinogen III oxidase	G127V	Deleterious	S
dhaM, b1198, ECK1186	3-AA-AT	Dihydroxyacetone kinase subunit M	V196A	Neutral	S
sspA, b3229, ECK3218	5-AA-AT	Stringent starvation protein A	Q24*	Deleterious	R
stpA, b2669, ECK2663	6-AA-AT	H-NS-like DNA-binding protein with RNA chaperone activity	R49S	Neutral	R
yhdW, b3268, ECK3255	8-AA-AT	Putative transport protein	C→A, pseudogene	NA	NA
rpoB, b3987, ECK3978	7-NA-AT, 8-NA-AT	RNA polymerase, β subunit	T135P	Deleterious	R
yqiB, b3033, ECK3024	7-NA-AT, 8-NA-AT	Predicted dehydrogenase	Q117K	Deleterious	S
metG, b2114, ECK2107	7-NA-AT	Methionyl-trna synthetase	R600P	Deleterious	R
cra /ilvH	4-AA-AT	Intergenic	C→T, intergenic	Deleterious	R
dxr/frr	8-NA-AT	Intergenic	T→A, intergenic	Deleterious	R
ynaE/pinR	7-NA-AT, 8-NA-AT	Intergenic	A→G, intergenic	Deleterious	R
yfjM /yfjL	4-AA-AT	Intergenic	(CATAGTGC) 6→7,inter-	Deleterious	R

			genic		
uspC/flhD	6-AA-AT	Intergenic	+4 bp, intergenic	Deleterious	R
ΔykfC, b0258, ECK0260	6-AA-AT	CP4-6 prophage; conserved protein	Deletion	Deleterious	S
ΔinsN, b0255, ECK0257	6-AA-AT	CP4-6 prophage; partial regulator of insertion element IS911A	Deletion	Deleterious	S
ΔinsI1, b0256, ECK0258	6-AA-AT	Transposase of IS30	Deletion	Deleterious	S
ΔperR b0254 ECK0256	6-AA-AT	Perr transcriptional regulator	Deletion	Deleterious	R
ΔykfA, b0253, ECK0255	6-AA-AT	Predicted GTP-binding protein	Deletion	Deleterious	R
ΔyafZ, b0252, ECK0254	6-AA-AT	CP4-6 prophage; conserved protein	Deletion	Deleterious	NA
ΔykfK, b4628, ECK4444	6-AA-AT	Pseudogene	Deletion	Deleterious	NA
ΔykfL, b4627, ECK4445	6-AA-AT	Pseudogene	Deletion	Deleterious	NA
ΔyafY, b0251, ECK0253	6-AA-AT	CP4-6 prophage; inner membrane lipoprotein	Deletion	Deleterious	NA
ΔykfB, b0250, ECK0252	6-AA-AT	Predicted protein	Deletion	Deleterious	NA
ΔykfF, b0249, ECK0251	6-AA-AT	Predicted protein	Deletion	Deleterious	NA
ΔyafX, b0248, ECK0250	6-AA-AT	Predicted protein	Deletion	Deleterious	NA
ΔykfG, b0247, ECK0249	6-AA-AT	Predicted DNA repair protein	Deletion	Deleterious	NA
ΔykfH, b4504, ECK0248	6-AA-AT	Predicted protein	Deletion	Deleterious	NA
ΔyafW, b0246, ECK0247	6-AA-AT	Antitoxin of the ykfi-yafw toxin-antitoxin pair	Deletion	Deleterious	R
ΔykfI, b0245, ECK0246	6-AA-AT	Toxin of the ykfi-yafw toxin-antitoxin system	Deletion	Deleterious	R
ΔykfN, *NA*	6-AA-AT	Pseudogene	Deletion	Deleterious	S
ΔthrW, b0244, ECK0245	6-AA-AT	Trna: trna^{thrw}	Deletion	Deleterious	R
ΔproA, b0243, ECK0244	6-AA-AT	Component of glutamate-5-semialdehyde	Deletion	Deleterious	S

		dehydrogenase			
ΔproB, b0242, ECK0243	6-AA-AT	Component of γ-glutamyl kinase	Deletion	Deleterious	S
ΔinsF1, b0299, ECK0299	2-AA-AT	IS3 element protein insf	Deletion	Deleterious	NA
ΔinsE1, b0298, ECK0298	2-AA-AT	IS3 element protein inse	Deletion	Deleterious	NA
ΔgatR, b2087, ECK2083	2-AA-AT	Negative DNA-binding transcriptional regulator of galactitol metabolism	Deletion	Deleterious	R
ΔyegS, b2086, ECK2082	2-AA-AT	Lipid kinase	Deletion	Deleterious	S
ΔyegR, b2085, ECK2081	2-AA-AT	Predicted protein	Deletion	Deleterious	NA
ΔyegZ, b2083, ECK2080	2-AA-AT	Predicted protein fragment	Deletion	Deleterious	NA
ΔogrK, b2082, ECK2079	2-AA-AT	DNA-binding transcriptional regulator, prophage P2 remnant	Deletion	Deleterious	R
ΔyegQ, b2081, ECK2077	2-AA-AT	Predicted peptidase	Deletion	Deleterious	NA
ΔyegP, b2080, ECK2076	2-AA-AT	Predicted protein	Deletion	Deleterious	NA
ΔbaeR, b2079, ECK2075	2-AA-AT	Baer transcriptional regulator	Deletion	Deleterious	R
ΔbaeS, b2078, ECK2074	2-AA-AT	Baes sensory histidine kinase	Deletion	Deleterious	R
ΔmdtD, b2077, ECK2073	2-AA-AT	Putative transport protein, major facilitator superfamily (MFS)	Deletion	Deleterious	S
ΔmdtC, b2076, ECK2072	2-AA-AT	Mdtabc-tolc multidrug efflux system - membrane subunit	Deletion	Deleterious	S
ΔmdtB, b2075, ECK2071	2-AA-AT	Mdtabc-tolc multidrug efflux system - membrane subunit	Deletion	Deleterious	S
wcaN, b2042, ECK2036	6-AA-AT	Predicted uridylyltransferase subunit with galu	-1 bp,	Deleterious	S
gltD, b3213, ECK3203	5-AA-AT, 6-AA-AT. 8-AA-AT, 5-AA-PT 6-AA-PT	Glutamate synthase, small subunit	N354D	Neutral	S
crr/ptsI	5-AA-PT	Intergenic	A→C	NA	R
rcnR/thiM	5-AA-PT, 6-AA-PT	Intergenic	A→G	NA	R
rph, b3643, ECK3633	3-AA-AT, 4-AA-AT, 6-AA-AT, ,	Coding	- 82 bp	Deleterious	S

	6-AA-PT				
rph/pyrE	5-AA-AT, 3-AA-PT, 4-AA-PT, 7-NA-AT, 8-NA-AT, 8-NA-PT	Intergenic and coding	-1 bp	Deleterious	S
wzxC/wcaK	8-AA-AT, 7-NA-PT, 8-NA-PT	Intergenic	+85 bp	NA	S
rpsK, b3297, ECK3284	5-AA-PT	30S ribosomal subunit protein S11	I116N	Neutral	S
cra, b0080, ECK0081	3-AA-PT, 4-AA-PT	DNA-binding transcriptional dual regulator	L191F	Neutral	R
fecR, b4292 ECK4282	6-AA-PT	Regulator for fec operon	A12G	Neutral	R
hflC, b4175, ECK4171	6-AA-PT	Regulator of ftsh protease	G268D	Neutral	R
mppA/pgrR	8-NA-PT	Intergenic	IS5 (–) +4 bp	NA	R

References:

1. Choi Y, Sims GE, Murphy S, Miller JR, Chan AP. Predicting the Functional Effect of Amino Acid Substitutions and Indels. PLoS ONE. 2012; 7: e46688.

2. Keseler I, Collado-Vides J, Santos-Zavaleta A, Peralta-Gil M, Gama-Castro S. EcoCyc: a comprehensive database of *Escherichia coli* biology. Nucleic Acids Res. 2011; 39: D583 - D590.
