# Supplementary material for: Experimental Evolution of Metabolic Dependency in Bacteria
Source: PLoS Genet. 2016 Nov 4;12(11):e1006364. doi: 10.1371/journal.pgen.1006364 (PMC5096674; doi:10.1371/journal.pgen.1006364)
Supplement: S3 Table — The genomic features are also illustrated in Fig 5. Strain identity code: X-Y-Z where X refers to a particular population (1–8), Y refers to the regime, i.e. AA = amino acid, or NA = no amino acid, and Z refers to the phenotype, i.e. either AT = auxotrophic or PT = prototrophic. (PDF) [file pgen.1006364.s006.pdf]

S3 Table. Genotypic and phenotypic characteristics of sequenced auxotrophic and prototrophic genotypes. The genomic features are also illustrated in Fig. 5. *Strain identity* code: X-Y-Z where X refers to a particular population (1-8), Y refers to the regime, i.e. AA = amino acid, or NA = no amino acid, and Z refers to the phenotype, i.e. either AT = auxotrophic or PT = prototrophic.

Strain identity	Genotype	Amino acid auxotrophies
**2-AA-AT**	*ptsP (M604R), yoaA (D121Y), insF1–mdtB (Δ 13,852 bp)*	Arg, Asn, Asp, Cys, Gly, His, Phe, Ser, Thr, Val
**3-AA-AT**	*hemF (G127V) , dhaM (V196A), rph (-82 bp)*	Ala, Arg, Asn, Asp, Cys, Gln, Glu, Gly, His, Ile, Leu, Lys, Met, Phe, Pro, Ser, Thr, Trp, Tyr, Val
**4-AA-AT**	*rph (-82 bp), yfjM / yfjL [(CATAGTGC)6→7], cra / ilvH (C→T)*	Arg, Asn, Asp, Cys, Gln, Gly, His, Phe, Ser & Thr
**5-AA-AT**	*rph / pyr (-1 bp), sspA (Q24*), gltD N354D , crr / ptsI (A→C)*	Arg, Asn, Asp, Cys, Gln, Gly, His, Phe, Ser, Thr, Val
**6-AA-AT**	*gltD (N354D), stpA (R49S), rcnR / thiM (A→G), wcaN (-1 bp), uspC / flhD (+4 bp), ykfC–proB (Δ13,518 bp);*	Pro
**8-AA-AT**	*yhdW (C→A), gltD (N354D), rcnR / thiM (A→G), wzxC / wcaK (+85 bp),*	Gly
**7-NA-AT**	*yqiB (Q117K), rpoB (T135P)*	Lys, Trp
**8-NA-AT**	*yqiB (Q117K), rpoB (T135P), metG (R600P), dxr/frr (T→A), ynaE/pinR (A→G)*	Lys, Trp
**3-AA-PT**	*rph / pyr (-1 bp), cra (L191F)*	NA
**4-AA-PT**	*rph / pyr (-1 bp), cra (L191F)*	NA
**5-AA-PT**	*rpsK (I116N), gltD (N354D), crr/ptsI (A→C),rcnR/thiM (A→G)*	NA
**6-AA-PT**	*rph (-82 bp), fecR* (A12G) *, hflC* (G268D)*, gltD (N354D), rcnR/thiM (A→G)*	NA
**7-NA-PT**	*wzxC/wcaK (*+85 bp*)*	NA
**8-NA-PT**	*rph / pyr (-1 bp), wzxC/wcaK (*+85 bp*), mppA/pgrR (IS5 +4 bp)*	NA
