# Supplementary material for: Experimental Evolution of Metabolic Dependency in Bacteria
Source: PLoS Genet. 2016 Nov 4;12(11):e1006364. doi: 10.1371/journal.pgen.1006364 (PMC5096674; doi:10.1371/journal.pgen.1006364)
Supplement: S4 Table — Abbreviations: Ara+/- = ability to use arabinose as a carbon source present/ absent, WT = wild type. (PDF) [file pgen.1006364.s007.pdf]

S4 Table. Strains used in this study. Abbreviations: $Ara^{+/-}$ = ability to use arabinose as a carbon source present/ absent, WT = wild type.

Strain	Genotype	Reference
Escherichia coli BW25113 Ara^{-}	*F-, ΔaraD-araB567, ΔlacZ4787::rrnB-3, $λ^{-}$, rph-1, ΔrhaD-rhaB568, hsdR514*	[1]
Escherichia coli BW25113 Ara^{+}	*F-, ΔaraD-araB567, ΔlacZ4787::rrnB-3, $λ^{-}$, rph-1, ΔrhaD-rhaB568, hsdR514, araA*	[2]
insF1- mdtB	*WT Ara^{-}, ΔinsF1- mdtB*	This study
ptsP	*WT Ara^{-}, ptsP*: M604R	This study
yoaA	*WT Ara^{-}*, yoaA: D121Y	This study
hemF	*WT Ara^{-}, hemF*: G127V	This study
sspA	*WT Ara^{-}, sspA*: Q24*	This study
stpA	*WT Ara^{-}, stpA*:R49S	This study
uspC / flhD	*WT Ara^{-}, uspC / flhD*: +4 bp	This study
wcaN	*WT Ara^{-}, wcaN*: -1 bp	This study
ykfC – proB	*WT Ara^{-}, ΔykfC - proB*	This study
yhdW	*WT Ara^{-}, yhdW*: C→A	This study
rpoB	*WT Ara^{-}, rpoB*:T135P	This study
yqiB	*WT Ara^{-}, yqiB*: Q117K	This study
ynaE / pinR	*WT Ara^{-}, ynaE / pinR*: A→G	This study

References:

1. Baba T, Ara T, Hasegawa M, Takai Y, Okumura Y, et al. Construction of *Escherichia coli* K-12 in-frame, single-gene knockout mutants: the Keio collection. Mol Sys Biol. 2006; 2:2006.0008.

2. D'Souza G, Waschina S, Pande S, Bohl K, Kaleta C, et al. Less is more: Selective advantages can explain the prevalent loss of biosynthetic genes in bacteria. Evolution. 2014; 68: 2559 - 2570.
